# Genome-Based Epidemiologic Analysis of VIM/IMP Carbapenemase-Producing *Enterobacter* spp., Poland

**DOI:** 10.3201/eid2908.230199

**Published:** 2023-08

**Authors:** Radosław Izdebski, Marta Biedrzycka, Paweł Urbanowicz, Dorota Żabicka, Marek Gniadkowski

**Affiliations:** Medicines Institute, Warsaw, Poland

**Keywords:** *Enterobacter*, VIM, Enterobacterales, *E. hormaechei*, ST90, ST89, Poland, bacteria, antimicrobial resistance

## Abstract

We sequenced all nonduplicate 934 VIM/IMP carbapenemase-producing Enterobacterales (CPE) reported in Poland during 2006–2019 and found ≈40% of the isolates (n = 375) were *Enterobacter* spp. During the study period, incidence of those bacteria gradually grew in nearly the entire country. The major factor affecting the increase was clonal spread of several *E. hormaechei* lineages responsible for multiregional and interregional outbreaks (≈64% of all isolates), representing mainly the pandemic sequence type (ST) 90 or the internationally rare ST89 and ST121 clones. Three main VIM-encoding integron types efficiently disseminated across the clone variants (subclones) with various molecular platforms. Those variants were predominantly *Pseudomonas aeruginosa*–derived In238-like elements, present with IncHI2+HI2A, IncFII+FIA, IncFIB, or IncN3 plasmids, or chromosomal genomic islands in 30 *Enterobacter* STs. Another prevalent type, found in 34 STs, were In916-like elements, spreading in Europe recently with a lineage of IncA-like plasmids.

In the past few decades, bacterial infections with limited therapeutic options have become a serious threat for medicine. This problem is primarily caused by antimicrobial resistance (AMR), which disseminates by clonal spread of resistant organisms and horizontal transmission of mobile genetic elements with AMR genes. Several taxa have been classified as main AMR pathogens, including *Klebsiella pneumoniae* and *Enterobacter* spp. of the order Enterobacterales (*1*), and carbapenemase-producing Enterobacterales (CPE) are among the most challenging multidrug-resistant organisms (*2*). Important carbapenemase types, metallo-β-lactamases (MBLs) of the families VIM and IMP, have been recorded in enterobacteria in Europe since 2001 (*3*), often in the Mediterranean region (*4*–*10*). The *bla*_VIM/IMP_ gene cassettes have usually been located in class 1 integrons, either assembled in *Pseudomonas* spp. and then transferred to Enterobacterales (*4*–*6*) or typical for Enterobacterales (*4*,*7*–*10*). The integrons have been carried by diverse plasmids with various replicons (*4*,*7*,*8*,*10*,*11*).

In Poland, VIM-type enzymes were originally identified in 2006 in *K. pneumoniae,* followed soon by *Enterobacter hormaechei* (*12*). Molecular analysis of all 121 VIM/IMP CPE isolates from 2006–2012 revealed high prevalence of *Enterobacter* spp. (≈53%) and relatively low contribution of *K. pneumoniae* (≈9%). *Enterobacter* spp. was dominated by *E. hormaechei* sequence type (ST) 90 and ST89, mostly with In238-like integrons of *Pseudomonas aeruginosa* origin. We describe the genomic analysis of all VIM/IMP *Enterobacter* spp. isolates in Poland during 2006–2019, in the context of all VIM/IMP CPE from that period, and international *Enterobacter* spp. genomes from public databases.

## Methods

### Study Design, Bacterial Isolates, Whole-Genome Sequencing, and Species Identification

The National Reference Centre for Susceptibility Testing conducts CPE surveillance in Poland, collecting isolates with basic patient, hospital ward, and isolate data. We tested the isolates by using CarbaNP (*13*) and phenotypic tests (*14*), and used PCRs for *bla*_NDM_-, *bla*_VIM_-, *bla*_IMP_-, *bla*_KPC_-, and *bla*_OXA-48_-like genes (*4*). A collection of 934 isolates from 246 hospitals in 117 cities were all nonduplicate VIM/IMP CPE confirmed during 2006–2019. We sequenced all those isolates by using MiSeq (Illumina, https://www.illumina.com), with de novo assemblies as described (*15*), and subjected them to species identification on the basis of average nucleotide identities by using FastANI 1.32 with a >95% cutoff (*16*). We further analyzed the largest group of 375 isolates of the genus *Enterobacter* from 145 hospitals in 76 towns. We also sequenced 9 selected isolates by using MinION (Oxford Nanopore Technologies, https://nanoporetech.com) (*15*). We performed hybrid assemblies by using Unicycler 0.4.8 (*17*).

### Molecular Typing and Comparative Genomic Analysis

We performed multilocus sequence typing (MLST) of all 375 *Enterobacter* spp. isolates (*18*) in silico by using mlst (https://github.com/tseemann/mlst). We performed the in-sample clonality single-nucleotide polymorphism (SNP) analysis for individual sequence types (STs) by using BioNumerics 7.6.3 (Applied Maths, https://www.applied-maths.com) and using index (i.e., initial) isolates of the STs as references. For the SNP-based phylogenetic analysis in the international context, we downloaded all (nonfiltered) 3,244 *Enterobacter* spp. genomes available in RefSeq (https://www.ncbi.nlm.nih.gov/refseq) as of June 6, 2022, and subjected them to MLST. We included isolates of the major STs (Appendix Table 1) in our analysis, which we performed by using Parsnp 1.5.4 (https://github.com/marbl/parsnp). We visualized the Parsnp-generated phylotrees by using iTOL (https://itol.embl.de).

### Acquired AMR Genes, Integrons, and Plasmids or Genomic Islands Carrying *bla*_VIM/IMP_ Genes

We detected acquired AMR genes by using ABRicate and the ResFinder database with 99.5% identity criterion (*19*) and profiled replicon types with PlasmidFinder 2.1 (*20*). We performed structural analysis and annotation of MBL-encoding integrons, plasmids, and genomic islands manually in Geneious Prime 2022.0.1 (Biomatters, https://www.geneious.com) by using BLASTn (https://blast.ncbi.nlm.nih.gov/Blast.cgi). We visualized plasmid and island structures by using BRIG (http://brig.sourceforge.net) and Easyfig 2.2.5. (http://mjsull.github.io/Easyfig).

### Nucleotide Sequence Accession Numbers

We submitted genomic data for the *Enterobacter* spp. isolates to the US National Center for Biotechnology Information (BioProject no. PRJNA877430). Plasmid sequences are available under the following GenBank accession numbers: p743A, OQ111274; p5955A, OQ111275; p7753A, OQ111276; p4969H, OQ111277; p5435N, OQ111278; p5713F, OQ111279; p6234F, OQ111280. Sequences of genomic islands are available under the following GenBank accession numbers: *Eh*GI3, OQ116783; *Eh*GI4, OQ116782,.

## Results

### Taxonomic Distribution of VIM/IMP-Type CPE in Poland

We collected 934 VIM/IMP CPE during 2006–2019 from 246 hospitals in 117 cities of all 16 regions of Poland (Appendix Figure 1, panel A). In annual numbers of cases, a gradual increase occurred, from a few cases during 2006–2008 up to 242 in 2019 (Appendix Table 2). We identified 9 genera, including *Enterobacter* (40.1%), *Klebsiella* (*K. pneumoniae* and *K. oxytoca* groups, 34.4%), *Citrobacter* (10.7%), *Escherichia* (9.2%), and *Serratia* (4.2%). The distribution of genera varied in time, including predominance of *Enterobacter* spp. and remarkable contribution of *K. oxytoca* during 2006–2013 (*12*) and still high prevalence of *Enterobacter* spp. but also a dynamic *K. pneumoniae* increase during 2014–2019 (Appendix Figure 1, panel B). Of note, annual numbers of *Enterobacter* spp. isolates grew at a roughly constant rate by the end of 2018, then escalating in 2019. VIM-type MBLs prevailed vastly (99.3%), whereas IMPs contributed marginally (0.7%). The 375 *Enterobacter* spp. isolates originated from 145 hospitals out of 76 towns and were recovered during various infections (64.3%), mainly of the urinary tract (31.5% of the infections) and wounds (28.6%), or from carriage (34.9%).

**Figure 1:**
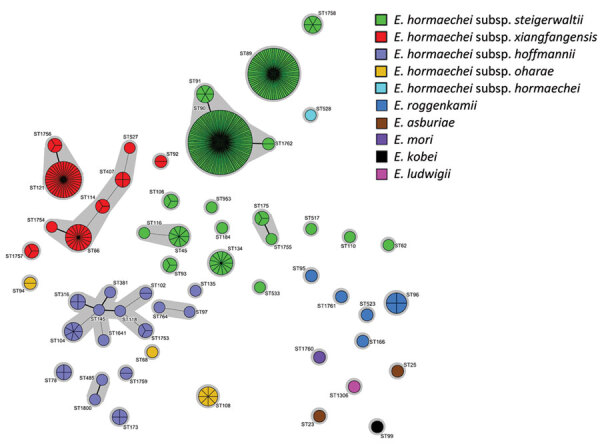
Population structure of *Enterobacter* spp. isolates identified in a genome-based epidemiologic analysis of VIM/IMP carbapenemase-producing *Enterobacter* spp., Poland, 2006–2019. The minimum-spanning tree was constructed on the basis of 7-loci multilocus sequence type data. Each circle represents 1 ST, and each fragment of a pie chart corresponds to 1 isolate. The size of a circle is proportional to the number of isolates of that ST. Connecting lines infer phylogenetic relatedness in terms of several allelic differences (thick solid line indicates a single-locus variant, thin solid line indicates a double-locus variant). ST, sequence type.

### Species and Clonality of *Enterobacter* spp.

We identified 6 species among the 375 *Enterobacter* isolates, largely *E. hormaechei* (362 [96.5%]) with 5 subspecies: *steigerwaltii* (n = 244), *xiangfangensis* (n = 71), *hoffmannii* (n = 35), *oharae* (n = 11) and *hormaechei* (n = 1) (Appendix Table 2). The remaining species were *E. roggenkampii* (8 [2.2%]), *E. asburiae* (2 [0.5%]), and *E. kobei*, *E. ludwigii*, and *E. mori* (1 [0.3%] each). We distinguished 56 STs (Table; Figure 1); 5 STs had >10 isolates each (258 [68.8%]): ST90 (117 [31.2%] of all *Enterobacter* spp.), ST89 (74 [19.7%]), ST121 (36 [9.6%]), ST66 (18 [4.8%]), and ST134 (13 [3.5%]). Isolates of closely related STs (single-locus variants) represented clonal groups (CGs) or clonal complexes (CCs) (Table; Figure 1).

**Table:** Clonal diversity of *Enterobacter* spp. with *bla*_VIM_ or *bla*_IMP_ genes, integrons, and SNP scores within individual genotypes, Poland, 2006–2019*

Species/subspecies and clonal status†	ST	*bla*_VIM/IMP_ gene	Integron‡	SNPs within the ST
*E. hormaechei* subsp. *steigerwaltii*, n = 244				
CG45, 13 SLVs	ST45, n = 9	*bla*_VIM–4_, n = 7	In238	0–8
		*bla*_VIM-1_, n = 2	In916	592
ST80–ST116–ST582, DLV of ST45	ST116, n = 1	*bla*_VIM-1_	**In2240**	–
ST62–ST421–ST1924	ST62, n = 1	*bla*_VIM-1_	In916	–
Singleton	ST89, n = 74	*bla*_VIM-1_, n = 48	In916	0–75
		*bla*_VIM-20_, n = 12	In1444	0–49
		*bla*_VIM-40_, n = 7	In1445	4–12
		*bla*_VIM-4_, n = 5	In238, n = 3	162–347
			In1654, n = 1	–
			**In2238**, n = 1	–
		*bla*_VIM-2_, n = 2	In1008	0
CC90, 10 SLVs	ST90, n = 117	*bla*_VIM-4_, n = 112	In238, n = 107	0–1,692
			238a, n = 5	36–61
		*bla*_VIM-4_; *bla*_IMP-19_, n = 2	In238a; **In2241**	3
		*bla*_VIM-1_, n = 3	In916, n = 1	–
			**In2240**, n = 1	–
			In237a, n = 1	–
CC90, SLV of ST90	ST91, n = 6	*bla*_VIM-1_, n = 4	In70, n = 3	4–73
			In916, n = 1	–
		*bla*_VIM-2_, n = 2	In1008	7
CC90, SLV of ST90	**ST1762**, n = 2	*bla*_VIM-4_, n = 2	In238	70
CG93, 11 SLVs	ST93, n = 3	*bla*_VIM-4_, n = 2	In238	60
		*bla*_VIM-2_, n = 1	In1008	–
CG106, 7 SLVs	ST106, n = 3	*bla*_VIM-1_	In916	0–1
ST110–ST226–ST656	ST110, n = 1	*bla*_VIM-1_	In916	–
ST134–ST1250–ST1901	ST134, n = 13	*bla*_VIM-4_, n = 11	In238, n = 9	6–24
			In238a, n = 2	4
		*bla*_VIM-1_, n = 2	In916	0
CG175, 3 SLVs	ST175, n = 3	*bla*_VIM-4_, n = 2	In238a	109
		*bla*_VIM-1_, n = 1	In916	–
CG175, SLV of ST175	**ST1755**, n = 1	*bla*_VIM-1_	In916	–
CG184, 5 SLVs	ST184, n = 1	*bla*_VIM-1_	In916	–
ST517–ST1443	ST517, n = 1	*bla*_VIM-20_	In1444	–
ST48–ST533–ST541	ST533, n = 1	*bla*_VIM-1_	In916	–
ST494–ST953	ST953, n = 1	*bla*_VIM-4_	In238a	–
ST535–ST537–ST1758	**ST1758**, n = 6	*bla*_VIM-1_, n = 6	In916	1–101
*E. hormaechei* subsp. *xiangfangensis*, n = 71				
CG66, 11 SLVs	ST66, n = 18	*bla*_VIM-1_	In916	0–404
CG66, SLV of ST66	**ST1754**, n = 1	*bla*_VIM-1_	In916	–
Singleton	ST92, n = 2	*bla*_VIM-4_	In238	1
CG114, 17 SLVs	ST114, n = 3	*bla*_VIM-4_, n = 2	In238	557
		*bla*_VIM-1_, n = 1	In916	–
CC121, 5 SLVs	ST121, n = 36	*bla*_VIM-1_, n = 24	In916	0–47
		*bla*_VIM-4_, n = 10	In238a, n = 6	2–10
			In238, n = 3	54–92
			In2016–like, n = 1	–
		*bla*_VIM-2_, n = 2	**In2242**	64
CC121, SLV of ST121	**ST1756**, n = 3	*bla*_VIM-1_	In916	5–15
ST407–ST511–ST552	ST407, n = 4	*bla*_VIM-1_, n = 2	In916	23
		*bla*_VIM-4_, n = 2	In238	87
Singleton	ST527, n = 1	*bla*_VIM-1_	In611-like	–
ST1348–ST1757	**ST1757**, n = 3	*bla*_VIM-1_	In916	5–14
*E. hormaechei* subsp. *hoffmannii*, n = 35				
CC78, 18 SLVs	ST78, n = 4	*bla*_IMP-19_, n = 2	**In2241**	41
		*bla*_VIM-1_, n = 1	In110	–
		*bla*_VIM-4_, n = 1	In238	–
ST97–ST754–ST761	ST97, n = 1	*bla*_VIM-1_	In916	–
ST102–ST343–ST1004	ST102, n = 2	*bla*_VIM-1_, n = 1	In916	–
		*bla*_VIM-4_, n = 1	In238a	–
ST104–ST345–ST1018	ST104, n = 7	*bla*_VIM-1_	In916	3–334
CG145, 5 SLVs; SLV of ST145	ST118, n = 1	*bla*_VIM-1_	In916	–
CG145, 12 SLVs; SLV of ST118, ST316, ST381	ST145, n = 1	*bla*_VIM-4_	In238	–
CG145, SLV of ST145	ST316, n = 4	*bla*_VIM-4_, n = 3	In238	3–72
		*bla*_VIM-1_, n = 1	In916	–
CG145, SLV of ST145	ST381, n = 1	*bla*_VIM-1_	In916	–
ST128–ST161–ST135	ST135, n = 1	*bla*_VIM-4_	In238	–
ST173–ST1619	ST173, n = 4	*bla*_VIM-1_, n = 2	In916	90
		*bla*_VIM-4_, n = 2	In238, n = 1	–
			In238a, n = 1	–
Singleton	ST485, n = 2	*bla*_VIM-4_	In238	216
ST363–ST764	ST764, n = 1	*bla*_VIM-1_	In916	–
CG1641, 4 SLVs	ST1641, n = 1	*bla*_VIM-1_	In916	–
ST961–ST1417–ST1753	**ST1753**, n = 3	*bla*_VIM-1_	In916	13–22
ST419–ST1759	**ST1759**, n = 2	*bla*_VIM-1_	In916	1
*E. hormaechei* subsp. *oharae*, n = 11				
CC68, 3 SLVs	ST68, n = 1	*bla*_VIM-4_	In238	–
ST94–ST471	ST94, n = 2	*bla*_VIM-4_	In238, n = 1	–
			In70, n = 1	–
CC108, 12 SLVs	ST108, n = 8	*bla*_VIM-2_	In1008	1–11
*E. hormaechei* subsp. *hormaechei*, n = 1				
ST262–ST269–ST528	ST528	*bla*_VIM-4_	In238	–
*E. roggenkampii*, n = 8				
CC96, 3 SLVs	ST96, n = 4	*bla*_VIM-4_	In238	37–49
ST95–ST515	ST95, n = 1	*bla*_VIM-4_	In238	–
ST166–ST433	ST166, n = 1	*bla*_VIM-1_	In916	–
ST523–ST823–ST1398	ST523, n = 1	*bla*_VIM-4_	In238	–
Singleton	**ST1761**, n = 1	*bla*_VIM-4_	In238	–
*E. asburiae*, n = 2				
Singleton	ST23, n = 1	*bla*_VIM-4_	In238	–
ST25–ST915–ST1407	ST25, n = 1	*bla*_VIM-4_	In238a	–
*E. kobei*, n = 1				
Singleton	ST99	*bla*_VIM-4_	In238	–
*E. ludwigii*, n = 1				
ST257–ST1306	ST1306	*bla*_VIM-1_	In916	–
*E. mori*, n = 1				
Singleton	**ST1760**	*bla*_VIM-4_	In238a	–

*Bold indicates STs originally identified in this study. CC, clonal complex; CG, clonal group; SLV, single locus variant; SNP, single-nucleotide polymorphism; ST, sequence type; –, not calculated. †Clonal status of an ST is defined against the entire multilocus sequence type database (https://pubmlst.org/ecloacae) as of September 20, 2022. STs are classified into CCs (separate groups of >4 closely related STs with a central genotype), CGs (groups of >4 closely related STs being parts of bigger straggly structures), groups of 2–3 closely related STs for which no central genotype may be currently distinguished, or as singletons. ‡New integron structures are bolded.

### *bla*_VIM_ and *bla*_IMP_ Genes and Their Integrons in *Enterobacter* spp.

We found 5 *bla*_VIM_ genes, primarily of the *bla*_VIM-1_ group (91.5% of all MBLs in *Enterobacter* spp.); most were *bla*_VIM-4_ (49.1%), *bla*_VIM-1_ (40.6%), and *bla*_VIM-40_ (1.9%) (Table). The *bla*_VIM-2_ group included *bla*_VIM-2_ (4.0%) and *bla*_VIM-20_ (3.4%), whereas all *bla*_IMP_s were *bla*_IMP-19_ (1.1%).

We characterized 16 integrons, including 4 new ones (Appendix Table 3). Elements of the In238 type prevailed (190 [50.4%]; 30 STs), carrying *bla*_VIM-4_ (In238/In238a)_,_ *bla*_VIM-40_ (In1445), or *bla*_VIM-1_ (In237a) genes. The second most prevalent In916 type (146 [38.7%]; 34 STs) had *bla*_VIM-1_. The *bla*_VIM-2_-like genes were located mostly in In1008-type integrons (26 [6.9%]; 5 STs), as *bla*_VIM-2_ (In1008) or *bla*_VIM-20_ (In1444). *bla*_IMP-19_ was in a new element In2241. We noticed temporal changes in the integron distribution; the incidence of In238s grew from 2009 (n = 6) to 2014 (n = 24) and then stabilized, whereas that of In916 rapidly increased from the original identification in 2014 (n = 9) to 2019 (n = 57).

### Epidemiology of Major *E. hormaechei* Clones and Multiregional and Interregional Outbreaks

**Figure 2:**
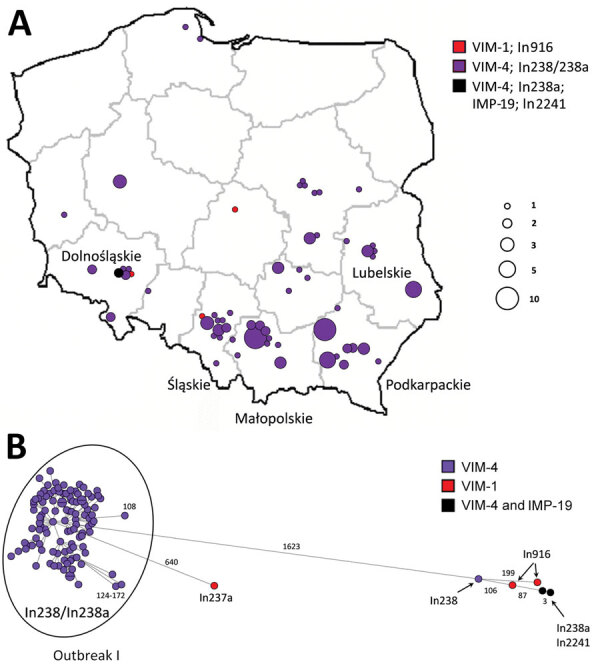
Geographic distribution and clonal analysis of *Enterobacter hormaechei* clonal complex 90 (ST90 and ST1762) in Poland, 2006–2019. A) Geographic distribution of the isolates; main administrative regions are labeled. Circles represent medical centers where the isolates were recorded. Sizes of the circles are proportional to numbers of cases of infection. B) SNP-based minimum-spanning tree of the isolates. Lengths of branches are related to numbers of SNPs between linked isolates. Numbers of SNPs are indicated above the branches or next to the dots. SNP, single nucleotide polymorphism; ST, sequence type.

The most widespread clone was *E. hormaechei* subsp. *steigerwaltii* ST90 (117 [31.2%]), recorded during 2009–2019 in 58 hospitals in 38 cities, mostly in southern regions (Figure 2; Appendix Table 4). Most of the 111 isolates with In238/In238a differed by 19–207 SNPs from the reference isolate (mean 71 SNPs) and formed a subclone (0–172 SNPs between closest relatives), likely resulting from multiregional expansion (outbreak I). We also classified 2 In238-carrying isolates of ST1762 (CC90) into this cluster (127–132 SNPs).

We observed *E. hormaechei* subsp. *steigerwaltii* ST89 (74 [19.7% of all isolates]) during 2006–2019 in 26 centers in 18 towns (Appendix Table 5, Figure 2). Most of the isolates (n = 67 [90.5% of ST89 isolates]) comprised 3 regional subclones with different integrons, representing outbreak II in Łódzkie (48 [0–75 SNPs between closest relatives]; In916), outbreak III in Wielkopolskie (12 [0–49 SNPs]; In1444), and outbreak IV in Kujawsko–Pomorskie (7 [4–12 SNPs]; In1445).

We identified *E. hormaechei* subsp. *xiangfangensis* CC121 isolates (ST121, 36 [9.6%]; ST1756, 3 [0.8%]) during 2014–2019 in 22 hospitals in 12 cities, mainly in the Mazowieckie and Łódzkie regions (Appendix Table 6, Figure 3). All those isolates were related to each other, with up to 84 SNPs with the reference (mean 46 SNPs); however, 2 outbreaks were distinguished based on the integron data: an interregional outbreak V (27 [0–46 SNPs between closest relatives]; In916) and a regional outbreak VI (6 [1–9 SNPs]; In238a).

Of the clones of lower incidence, ST66 and ST1754 (CG66; n = 19) were split into 2 genetically and geographically separated subclones (0–17 and 0–23 SNPs within the groups [404 SNPs between them]; both with In916), likely representing an interregional outbreak VII and a regional outbreak VIII (Appendix Table 7, Figure 4). ST134 (n = 13) showed variety as well, with a cluster of related organisms (9 [6–24 SNPs]; In238) arising from an apparent regional outbreak IX (Appendix Table 8, Figure 5).

### Phylogeny and International Context of Major *E. hormaechei* Clones

The clonal analysis of all 3,244 *Enterobacter* spp. genomes in RefSeq (as of June 6, 2022) revealed 546 STs; 61 STs were represented by >10 records. Out of the major VIM-positive clones in Poland, only ST90 and ST66 were among the 10 most numerous STs. Otherwise, the prevalent RefSeq clones were either not present (e.g., ST171 and ST133) or marginal (e.g., ST78 and ST114). However, the RefSeq genomes were unfiltered, which could have affected some of the observations. The phylogenetic analysis of 46 international ST90 genomes revealed 2 main clades and most of the 117 isolates in Poland, including outbreak I, belonged to a branch with several carbapenemase-free isolates from the United Kingdom, France, Portugal, and Brazil (Appendix Figure 6).

ST89 was represented in RefSeq only by 2 isolates in Germany (1 with GIM-1) and 24 NDM-1–positive isolates in Poland during 2017–2020, which we analyzed in a previous study (*21*). Therefore, the phylotree comprised 100 isolates, including 98 from Poland (Appendix Figure 7), and consisted of 2 major lineages, each split then into multiple branches, correlating with the regional distribution of the isolates, regardless of their MBL content. The first lineage contained all of the VIM outbreak II isolates in Łódzkie plus a cluster of related NDM isolates from a neighboring area. The second lineage was divided into 2 major branches, 1 of which comprised the VIM outbreak IV in Kujawsko–Pomorskie and a large NDM epidemic from the adjacent region of Mazowieckie. The other branch contained mainly isolates from western Poland, including the VIM outbreak III from Wielkopolskie. Consistently, the 2 isolates in Germany were also located on the latter branch.

Only 7 ST121 genomes were present in RefSeq; the 36 VIM isolates in Poland, including outbreaks V–VI, formed 1 of 2 main lineages together with isolates from Brazil, Uganda, Morocco, Germany, and Poland (NDM) (*21*) (Appendix Figure 8). A total of 51 international ST66 isolates formed 2 lineages; 8 isolates in Poland of the outbreak VII belonged, primarily, to the lineage with isolates from Spain, France, and Germany mainly, whereas 10 outbreak VIII isolates clustered within the second lineage of more global character (Appendix Figure 9). ST134 records were sporadic in RefSeq (n = 9), and the 13 isolates in Poland, including outbreak IX, were located within 1 lineage together with single isolates from the United States, Lebanon, and Iran (Appendix Figure 10).

### Resistomes

The resistome analysis demonstrated a large number and a variety of acquired AMR genes (6–27 genes per isolate; mean 15.8) (Appendix Table 9), in addition to the natural *Enterobacter* spp. *ampC* cephalosporinase genes. Their exact numbers could be specified only for the 9 MinION-sequenced genomes because some genes were in multiple copies in individual isolates (Appendix Table 10). The diversity of resistomes (AMR gene types and numbers) was common across and within the epidemic subclones; for some of those, the only stable AMR genes (i.e., present in all isolates of a subclone) were those in the MBL integrons. For instance, the ST90 isolates of the outbreak I had 67 AMR gene profiles, and ST89 isolates of the outbreak II had 33 AMR gene profiles. Along with *bla*_VIM/IMP_s, most of the isolates had genes coding for extended-spectrum β-lactamases (*bla*_SHV_ and *bla*_CTX-M_ types, *bla*_GES-7_, and *bla*_PER-2_) or acquired AmpC-like cephalosporinases (*bla*_CMY-83_, *bla*_DHA-1_, and *bla*_FOX-20_). Along with various aminoglycoside-modifying enzyme genes, numerous isolates had the 16S rRNA methylase gene *armA*, inactivating all aminoglycosides. Different variants of fluoroquinolone-resistance genes *qnrA*/*B*/*E*/*S* were commonly represented; 65 isolates contained the *mcr-9.1* colistin-resistance gene.

### Plasmids Harboring *bla*_VIM_ Genes

We identified 44 plasmid replicon types with 1–8 replicons per organism. The most frequent replicons were IncHI2 (n = 237), IncHI2A (n = 232), IncA (n = 165), IncFII (n = 140), and IncFIA (n = 116). Replicon profiles remarkably varied both between and within the subclones (Appendix Table 11). Long-read sequencing revealed the plasmid content, and the replicon and AMR gene distribution between the plasmids in 7 isolates representing the main epidemic subclones: ST90–In238 (n = 2; outbreak I), ST89–In916 (outbreak II), ST121–In916 (outbreak V), ST121–In238a (outbreak VI), ST66–In916 (outbreak VII), and ST134–In238 (outbreak IX) (Appendix Table 10). We performed the structural analysis on the plasmids with *bla*_VIM_-harboring integrons.

In the 4 isolates with In238/In238a, including the 2 ST90–In238 representatives, the integrons were on 4 different plasmids. In 1 of those (isolate 4969–09), In238 was on an IncHI2+HI2A plasmid (p4969H; ≈261 kb), related to numerous others from *Enterobacterales* worldwide (91%–95% coverage; ≈100% identity), occasionally with *bla*_IMP/VIM_ genes (Appendix Figure 11). One such plasmid from the Czech Republic, p51929_MCR_VIM (93% coverage; ≈100% identity), also contained In238 (*22*). The second ST90–In238 isolate (6234–09) had that integron on a plasmid with unique FII and FIA replicons (p6234F; ≈91 kb); FII was of some similarity to pECL_A (≈83%) (*23*) and FIA to R27 (≈84%) (*24*). The IncFII+FIA scaffold matched 9 GenBank records well (>60% coverage, >98% identity) (Appendix Figure 12). Of note, in p4969H and p6234F, the In238 integron was located in novel, almost identical Tn*21*-like transposons Tn*7536*, similar to Tn*1696* (*25*) (Appendix Figure 13).

The ST121–In238a isolate (5713–17) had In238a on an IncFIB-like plasmid (p5713F; ≈120 kb), with the replicon similar to pB171 (≈91%) (*26*), homologous to 8 *bla*_VIM_-negative records (80%–90% coverage; ≈100% identity) (Appendix Figure 14). Last, in the ST134–In238 isolate (5435–13) the integron resided on an IncN3-like plasmid (p5435N; ≈46 kb), matching several records (89% coverage; ≈100% identity), including some with *bla*_IMP/VIM_ genes (Appendix Figure 15). The In238-type integrons in p5713F and p5435N were not located in Tn*21*-like transposons.

In the 3 isolates with In916: ST89 (7753–18), ST121 (743–14) and ST66 (5955–16), the integron resided on IncA plasmids (p7753A, ≈162 kb; p743A, ≈170 kb; and p5955A, ≈154 kb). Those isolates were highly related to each other and to 9 In916-carrying IncA plasmids (84%–96% coverage, ≈100% identity), including 5 from Italy (different *Enterobacterales*) (*7*) and 1 from Poland (*K. pneumoniae*) (*27*) (Appendix Figure 16). The plasmids varied mostly by rearrangements within the AMR region containing an IS*26*–*bla*_SHV-12_–In916–IS*26* module (≈37.8-≈51.8 kb). This region in p743A was almost identical to plasmids pGB_VIM and pGA_VIM from Italy (*7*) (Appendix Figure 17).

### Genomic Islands with *bla*_VIM_ Genes

An isolate representing the epidemic subclone ST89–In1445 (8770–11; outbreak IV) had a new genomic island *Eh*GI3 with the *bla*_VIM-40_ gene, and the isolate of the clone ST89–In1444 (2944–06; outbreak III) had another new genomic island with *bla*_VIM-20_. *Eh*GI3 (≈94.6 kb), inserted into the tRNA^Gly^ gene, was a *clc*-like integrative and conjugative element (ICE) (41% coverage and ≈87% identity with the *clc* reference [*28*]), similar to ICEs found mainly in pseudomonads (*29*) (Appendix Figure 18). *Eh*GI4 (≈71.1 kb) was a mosaic region flanked by 2 IS*26* copies with direct repeats, carrying In1444 and multiple AMR genes (e.g., *armA*).

## Discussion

We describe VIM/IMP CPE in Poland, which markedly increased in recent years after a period of rather low prevalence. During 2017–2019, the annual VIM/IMP CPE numbers recorded by the National Reference Centre for Susceptibility Testing (n = 545) were comparable with KPC (n = 686) or OXA-48 (n = 383) producers but far behind NDM organisms (n>6,000 [https://www.korld.nil.gov.pl]) (*12*,*14*,*15*,*21*,*30*). Among all carbapenemase-producing *Enterobacter* spp., the organisms with VIM/IMP-like enzymes were the predominant group (59.4%). The leading position of *Enterobacter* spp. among VIM/IMP CPE was maintained for all years of the study; however, the dynamic spread of *K. pneumoniae* in more recent years has notably changed the species composition. A substantial role of *Enterobacter* spp. among VIM CPE has been observed also in other countries of Europe (*8*,*11*).

The successful dissemination of VIM-producing *Enterobacter* spp. in Poland has depended largely on several epidemic subclones of *E. hormaechei* ST90, ST89, and ST121 lineages, responsible for multiregional and interregional outbreaks I–VI (≈63% of all isolates). ST90 is a global clone, often reported with various carbapenemases (*11*). Its population in Poland has been dominated by the ST90–In238/In238a subclone, and since 2009 it has been expanding over a large territory (outbreak I). On the contrary, ST89 seems to be a local lineage, having been reported mostly in Poland with various VIMs, OXA-48, or NDM-1 so far. However, its repeated identification with GIM-1 in Germany indicates broader spread in central Europe (*21*,*31*,*32*). 

The ST89 VIM-producing isolates in Poland were clustered into 3 regional subclones, ST89–In916, ST89–In1444, and ST89–In1445 (outbreaks II–IV), closely related to the previously described ST89 NDM-1 subclones from the same or neighboring areas (*21*). This finding indicates that ST89 has produced a series of regional sublineages, acquiring and then disseminating with different AMR genes. The epidemiology of ST121 has been unclear. According to RefSeq, it appears to be nonprevalent, although present broadly in the world. In Poland, it has spread extensively, acquiring several VIM integrons and causing major regional outbreaks (V-VI).

The second essential factor of the VIM-producing *Enterobacter* spp. expansion in Poland has been the horizontal transmission of 3 major VIM integron types. The In238 type with *bla*_VIM-1_-like genes and In1008 type with *bla*_VIM-2_-like genes formed 2 evolving families of elements, with individual variants differing by mutations in *bla*_VIM_ cassettes, and by 3′-termini of these in the case of In238 (specific 169bp repeats in some variants) (*12*,*33*). Both types were found originally in *P. aeruginosa* in Poland in 1998 (In238) (*33*) and 2001 (In1008) (*34*) and most likely were transmitted to Enterobacterales during 2006–2009 (*12*). However, In238 variants have been observed more broadly in central and southern Europe (*22*,*35*–*37*). The third major integron type, Enterobacterales-specific In916, has been recorded since the early 2010s in Spain, Italy, and France (*4*,*8*,*11*,*38*), and in Poland it has spread since at least 2013 (R. Izdebski and M. Gniadkowski, unpub. data). All those integron types have been acquired by *E. hormaechei* at the beginning of their dissemination in Enterobacterales in Poland with various molecular platforms.

In our previous study, the 2006–2012 predominant In238-type integrons in *E. hormaechei* ST89 and ST90 were assigned to IncHI2, PCR-nontypeable (largely), or IncM plasmids (*12*). We long-read sequenced 2 ST90–In238 isolates, representing outbreak I, in this study and found them to have In238 on the IncHI2+HI2A or IncFII+FIA (previously nontypeable) plasmids, suggesting exchange between them. Given that the integron was located within almost identical Tn*21*-like transposons (Tn*7536*) in both plasmids, those might have been responsible for the inter-plasmid transfer. However, the 2 remaining long-read sequenced ST121 and ST134 isolates with In238/In238a had these integrons on yet other plasmids, IncFIB (ST121) and IncN3 (ST134), and not in a transposonic context. This finding indicates that acquisition and circulation of the In238-like elements among 36 STs of *Enterobacter* spp. in Poland have been multifactorial and complex phenomena. Regarding acquisition, an interesting case was provided by the ST89 isolate with the In238-like integron In1445, located within the *clc*-type ICE *Eh*GI3. In238 variants have been frequent in VIM-producing *P. aeruginosa* (*39*) and *P. putida* in Poland (*40*), being usually chromosomal in those. *Eh*GI3 turned out to be almost identical to an ICE in 1 of the *P. putida* group isolates, indicating exchange of such elements between pseudomonads and Enterobacterales (P. Urbanowicz, M. Gniadkowski, unpub. data).

On the other hand, the proliferation of In916 seems to be relatively clear. In Europe, this integron has been associated with IncA, IncFII_K_, IncHI2, IncN, or PCR-nontypeable plasmids (*4*,*7*,*8*), and in our study isolates, it has entirely correlated with the IncA plasmids. A close relatedness between the In916-carrying IncA plasmids in Poland and Italy was proved, which together with high conjugative potential (*7*) have explained their spread on a large geographic scale. As in Italy (*7*) and France (*8*), rapid dissemination of these plasmids in Poland since 2013–2014 has contributed to the increase in VIM-producing Enterobacterales and *Enterobacter* spp., making In916 the most prevalent integron in 2019 (≈63%). The In916-carrying IncA plasmids occurred in 30 *Enterobacter* STs, including ST89, CC121 and CG66 subclones of 4 regional outbreaks, revealing that both the horizontal and clonal spread contributed to their recent proliferation.

Our study has shown the epidemiology of VIM-producing *Enterobacter* spp. during 14 years of VIM CPE surveillance in Poland, substantially updating the previous report (*12*). The results enable the precise definition of several *E. hormaechei* subclones of a remarkable epidemic potential, responsible for a series of territorial outbreaks, and enable the characterization of the main molecular platforms transmitting integrons with *bla*_VIM_ genes in *Enterobacter* populations. The study revealed several factors specific for Poland or central Europe, namely the prominent role of apparently rare *E. hormaechei* clones (ST89 or ST121), peculiar integrons of pseudomonadal origins (In238 and In1008 types), and unique VIM-encoding plasmids (IncFII+FIA with In238). We have also demonstrated some cosmopolitan elements, such as the global status of the epidemic ST90 clone and pan-Europe dissemination of In916-carrying IncA-like plasmids. All these observations indicate that AMR VIM-producing *E. hormaechei* and the VIM-encoding plasmids create an epidemiologic danger for hospital environments throughout Europe that clinicians and infection control specialists should be aware of. 

Appendix Additional information about genome-based epidemiologic analysis of VIM/IMP carbapenemase-producing *Enterobacter* spp., Poland.
