# Supplementary material for: Genome-Based Epidemiologic Analysis of VIM/IMP Carbapenemase-Producing Enterobacter spp., Poland
Source: Emerg Infect Dis. 2023 Aug;29(8):1618–26. doi: 10.3201/eid2908.230199 (PMC10370858; doi:10.3201/eid2908.230199)
Supplement: Appendix — Additional information about genome-based epidemiologic analysis of VIM/IMP carbapenemase-producing Enterobacter spp., Poland. [file 23-0199-Techapp-s1.pdf]

Article DOI: https://doi.org/10.3201/eid2908.230199

EID cannot ensure accessibility for supplementary materials supplied by authors. Readers who have difficulty accessing supplementary content should contact the authors for assistance.

Genome-Based Epidemiologic Analysis of VIM/IMP Carbapenemase-Producing *Enterobacter* spp., Poland

Appendix

Appendix Table 1. RefSeq assembly numbers, countries and STs of international *Enterobacter* spp. genomes used in phylogenetic analyses.

RefSeq assembly numbers	Country code	Country name	ST
GCF_000770745.2	AU	Australia	ST90
GCF_002416795.1	ZA	South Africa	ST90
GCF_002334585.1	JP	Japan	ST90
GCF_002237465.1	AU	Australia	ST90
GCF_002334485.1	JP	Japan	ST90
GCF_003175745.1	JP	Japan	ST90
GCF_002334605.1	JP	Japan	ST90
GCF_013403065.1	FR	France	ST90
GCF_020526705.1	BR	Brazil	ST90
GCF_002923275.1	JP	Japan	ST90
GCF_900076435.1	GB	Great Britain	ST90
GCF_900076635.1	GB	Great Britain	ST90
GCF_900558595.1	GB	Great Britain	ST90
GCF_001525015.1	AU	Australia	ST90
GCF_001472575.1	GR	Greece	ST90
GCF_900077985.1	GB	Great Britain	ST90
GCF_018421195.1	DE	Germany	ST90
GCF_900076885.1	GB	Great Britain	ST90
GCF_013403105.1	FR	France	ST90
GCF_002334565.1	JP	Japan	ST90
GCF_002785805.1	CN	China	ST90
GCF_013403095.1	FR	France	ST90
GCF_900076465.1	GB	Great Britain	ST90
GCF_015209005.1	ZA	South Africa	ST90
GCF_900076555.1	GB	Great Britain	ST90
GCF_018447355.1	DE	Germany	ST90
GCF_002334545.1	JP	Japan	ST90
GCF_001475405.1	RO	Romania	ST90
GCF_001653625.1	PT	Portugal	ST90
GCF_002334525.1	JP	Japan	ST90
GCF_015208945.1	ZA	South Africa	ST90
GCF_900077715.1	GB	Great Britain	ST90
GCF_002417315.1	ZA	South Africa	ST90
GCF_900075615.1	GB	Great Britain	ST90
GCF_900076495.1	GB	Great Britain	ST90
GCF_900536495.1	FR	France	ST90
GCF_900076825.1	GB	Great Britain	ST90
GCF_015208805.1	ZA	South Africa	ST90
GCF_002334505.1	JP	Japan	ST90
GCF_001526025.1	EC	Ecuador	ST90
GCF_900076765.1	GB	Great Britain	ST90
GCF_022551835.1	TW	Taiwan	ST90
GCF_900076525.1	GB	Great Britain	ST90
GCF_008931785.1	GB	Great Britain	ST90
GCF_002740875.1	RO	Romania	ST90
GCF_002510085.1	ZA	South Africa	ST90

RefSeq assembly numbers	Country code	Country name	ST
GCF_018420435.1	DE	Germany	ST89
GCF_900497145.1	DE	Germany	ST89
GCF_900076335.1	GB	Great Britain	ST66
GCF_022685505.1	ES	Spain	ST66
GCF_001524975.1	CA	Canada	ST66
GCF_015701095.1	US	USA	ST66
GCF_022685645.1	ES	Spain	ST66
GCF_003289405.1	FR	France	ST66
GCF_003289795.1	FR	France	ST66
GCF_016428335.1	FR	France	ST66
GCF_016428325.1	FR	France	ST66
GCF_022685705.1	ES	Spain	ST66
GCF_013169535.1	CO	Colombia	ST66
GCF_012328865.1	FR	France	ST66
GCF_022685625.1	ES	Spain	ST66
GCF_009832255.1	US	USA	ST66
GCF_012328725.1	FR	France	ST66
GCF_013169525.1	CO	Colombia	ST66
GCF_022685745.1	ES	Spain	ST66
GCF_900076295.1	GB	Great Britain	ST66
GCF_019800365.1	DE	Germany	ST66
GCF_019837105.1	CN	China	ST66
GCF_012328705.1	FR	France	ST66
GCF_003289305.1	FR	France	ST66
GCF_022685685.1	ES	Spain	ST66
GCF_022685565.1	ES	Spain	ST66
GCF_020676035.1	NG	Nigeria	ST66
GCF_022685775.1	ES	Spain	ST66
GCF_012328715.1	FR	France	ST66
GCF_014190075.1	VN	Vietnam	ST66
GCF_003977165.1	EG	Egypt	ST66
GCF_016428365.1	FR	France	ST66
GCF_001473015.1	TW	Taiwan	ST66
GCF_900558375.1	GB	Great Britain	ST66
GCF_002208275.1	AU	Australia	ST66
GCF_002740595.1	TG	Togo	ST66
GCF_022685545.1	ES	Spain	ST66
GCF_004405115.1	CN	China	ST66
GCF_019448675.1	CN	China	ST66
GCF_900076615.1	GB	Great Britain	ST66
GCF_012328685.1	FR	France	ST66
GCF_012328885.1	FR	France	ST66
GCF_016428435.1	FR	France	ST66
GCF_019448715.1	CN	China	ST66
GCF_900076015.1	GB	Great Britain	ST66
GCF_004181915.1	FR	France	ST66
GCF_900538115.1	GB	Great Britain	ST66
GCF_021457515.1	TW	Taiwan	ST66
GCF_018447035.1	DE	Germany	ST66
GCF_019797615.1	DE	Germany	ST66
GCF_022685125.1	ES	Spain	ST66
GCF_022685515.1	ES	Spain	ST66
GCF_019797725.1	DE	Germany	ST66
GCF_018422515.1	DE	Germany	ST121
GCF_002154935.1	BR	Brazil	ST121
GCF_023060195.1	BR	Brazil	ST121
GCF_020922915.2	PL	Poland	ST121
GCF_014842835.1	PK	Pakistan	ST121
GCF_001472215.1	MA	Morocco	ST121
GCF_003968685.1	UG	Uganda	ST121
GCF_014903845.1	US	USA	ST134
GCF_000957655.1	US	USA	ST134
GCF_020567475.1	IR	Iran	ST134
GCF_900177445.1	-	-	ST134
GCF_900075455.1	GB	Great Britain	ST134
GCF_900447525.1	-	-	ST134
GCF_900075315.1	GB	Great Britain	ST134
GCF_011030345.1	LB	Lebanon	ST134
GCF_003444755.1	CN	China	ST134

Appendix Table 2. Annual taxa distribution of VIM/IMP-producing Enterobacterales in Poland, 2006-2019

genus	groups/species/subspecies	Year														Total
		2006	2007	2008	2009	2010	2011	2012	2013	2014	2015	2016	2017	2018	2019	
Enterobacter	*E. hormaechei* subsp. *steigerwaltii*	1	-	-	9	13	16	18	19	24	16	31	26	25	46	244
	E. hormaechei subsp. *xiangfangensis*	-	-	-	-	2	-	-	-	6	5	2	16	14	26	71
	E. hormaechei subsp. *hoffmannii*	-	-	-	-	-	-	1	-	3	2	5	5	5	14	35
	E. hormaechei subsp. *oharae*	-	-	-	-	-	-	1	-	-	7	-	2	-	1	11
	E. hormaechei subsp. *hormaechei*	-	-	-	-	-	-	-	-	-	-	-	-	-	1	1
	E. roggenkampii	-	-	-	-	-	-	2	-	1	1	1	-	-	3	8
	E. asburiae	-	-	-	-	-	-	-	-	-	-	1	-	1	-	2
	E. kobei	-	-	-	-	-	1	-	-	-	-	-	-	-	-	1
	E. ludwigii	-	-	-	-	-	-	-	-	-	-	-	-	1	-	1
	E. mori	-	-	-	-	-	-	-	-	-	1	-	-	-	-	1
	Total	**1**	**-**	**-**	**9**	**15**	**17**	**22**	**19**	**34**	**32**	**40**	**49**	**46**	**91**	**375**
Klebsiella	*K. pneumoniae* group	1	0	1	3	0	3	3	2	20	8	16	32	61	65	215
	K. oxytoca group				4	5	6	7	8	6	7	14	17	13	19	106
Citrobacter		-	-	-	-	-	1	1	3	9	6	8	14	29	29	100
Escherichia		-	-	-	-	-	-	-	2	7	4	11	17	13	32	86
Serratia		-	5	-	5	3	2	5	1	3	2	2	4	3	4	39
Morganella		-	-	-	-	-	-	-	-	-	1	1	1	3	1	7
Proteus		-	-	-	-	-	-	-	-	-	3	-	-	-	-	3
Leclercia		-	-	-	-	-	-	-	-	-	-	1	-	1	-	2
Hafnia		-	-	-	-	-	-	-	-	-	-	-	-	-	1	1
	Total	**2**	**5**	**1**	**21**	**23**	**29**	**38**	**35**	**79**	**63**	**93**	**134**	**169**	**242**	**934**

Appendix Table 3. VIM/IMP-encoding class 1 integrons identified in the *Enterobacter* study isolates

Integron type	Integron variant[a]	Gene cassette array	Number of STs	Country, year and species of the first identification[b, c]	GenBank entry
with *bla*$_{VIM-1}$-like genes					
In238 (n=190)	In238 (n=160)	5'CS_*aacA4*_*bla*$_{VIM-4rpt}$_3'CS	25	Poland, 1998, *P. aeruginosa*<br>Poland, 2008, *K. pneumoniae*<br>Poland, 2009, *E. hormaechei*	AJ585042/AY702100[d]
	In238a (n=22)	5'CS_*aacA4*_*bla*$_{VIM-4}$_3'CS	9	Poland, 2009, *E. hormaechei*	JQ003906 (Hungary 2010)
	In1445 (n=7)	5'CS_*aacA4*_*bla*$_{VIM-40rpt}$_3'CS	1	Poland, 2011, *E. hormaechei*	MF678585
	In237a[e] (n=1)	5'CS_*aacA4*_*bla*$_{VIM-1}$_3'CS	1	Poland, 2019, *K. oxytoca*<br>Poland, 2019, *E. hormaechei*	OQ116826
In916 (n=146)	In916 (n=144)	5'CS_*bla*$_{VIM-1}$_*aacA4*_*aphA15*_*aadA1*_*catB2*_3'CS	33	Spain, before 2014, *E. coli*<br>Poland, 2013, *E. coli* & *C. freundii*<br>Poland, 2014, *E. hormaechei*	KF856617
	**In2240** (n=2)	5'CS_*bla*$_{VIM-1}$_*aacA4*_*aphA15f*_*aadA1*_*catB2*_3'CS	2	Poland, 2015, *E. hormaechei*	OQ116829
In70 (n=4)	In70	5'CS_*bla*$_{VIM-1}$_*aacA4*_*aphA15*_*aadA1*_3'CS	2	Italy, 1997, *P. aeruginosa*<br>Poland, 2011, *E. hormaechei*	AJ969235
In1654 (n=1)	In1654	5'CS_*bla*$_{VIM-4rpt}$_3'CS	1	Poland, 2010, *P. aeruginosa*<br>Poland, 2014, *K. pneumoniae*<br>Poland, 2019, *E. hormaechei*	MW595328
In110 (n=1)	In110	5'CS_*bla*$_{VIM-1}$_*aacA4*_*aadA1*_3'CS	1	Italy, 1999, *P. putida*<br>Poland, 2006, *P. aeruginosa*<br>Poland, 2016, *E. hormaechei*	AJ439689
In2238 (n=1)	**In2238**	5'CS_*bla*$_{VIM-4rpt}$_*bla*$_{OXA-2}$_*aacA4*_3'CS	1	Poland, 2016, *E. hormaechei*	OQ116827
In611 (n=1)	In611-like	5'CS_*bla*$_{VIM-1}$_*aacA4*_*aacC1*_*gcuP*_*gcuQ*_Δ*aadA1*	1	Poland, 2018, *E. hormaechei*	-
In2016 (n=1)	In2016-like[f]	5'CS_*bla*$_{VIM-4rpt}$_*bla*$_{OXA-10}$_Δ3'CS	1	Poland, 2019, *E. hormaechei*	-
with *bla*$_{VIM-2}$-like genes					
In1008 (n=26)	In1008 (n=13)	5'CS_*bla*$_{VIM-2}$_*aacA4*_3'CS	4	Poland, 2001, *P. aeruginosa*<br>Poland, 2007, *S. marcescens*<br>Poland, 2009, *E. hormaechei*	AM087408
	In1444 (n=13)	5'CS_*bla*$_{VIM-20}$_*aacA4*_3'CS	2	Poland, 2006, *E. hormaechei*	MF678584
In2242 (n=2)	**In2242**	5'CS_*bla*$_{VIM-2}$_*aacC11b*_*aadA6-10*_3'CS	1	Poland, 2017, *E. hormaechei*	OQ116831
with *bla*$_{IMP}$-like genes					
In2241 (n=4)	**In2241**	5'CS_*bla*$_{IMP-19}$_*aacA4*_*aadA1b*_*catB2*_3'CS	2	Poland, 2017, *E. hormaechei*	OQ116830

[a] – new integrons are indicated in bold style.
[b] – when the first report was from another country, then it is followed by the first Polish case(s); if the first Polish record was from non-Enterobacterales and/or non-*Enterobacter* Enterobacterales, it is then followed by the first Polish Enterobacterales and *Enterobacter*, respectively.
[c] – date of isolation of the first Polish organism with a given integron may be earlier than that of the first isolate reported ever in another country.
[d] – the original In238 record (AJ585042) contains a 2 nt error in the *bla*$_{VIM-4}$ coding sequence; the subsequent *P. aeruginosa* In238 entry from Hungary from 2003 has been provided.
[e] – In237a differs from the In237-like element, reported in Greece and Poland (GenBank acc. No. AY152821; Scoulica EV et al. Diagn Microbiol Infect Dis. 2004;48:167-72; Izdebski R et al J Antimicrob Chemother. 2018;73:2675-81), by having no 3'-terminal 169bp tandem repeat in the *bla*$_{VIM-1}$ gene cassette.
[f] – In2016-like differs from In2016, reported originally in *P. aeruginosa* in Poland (GenBank acc. No. MW595340; Urbanowicz P et al. J Antimicrob Chemother. 2021;76:2273-84), by having the 3'-terminal 169bp tandem repeat in the *bla*$_{VIM-4}$ gene cassette.

Appendix Table 4. SNP scores between *E. hormaechei* subs. *steigerwaltii* CC90 (ST90 and ST1762) isolates[a,b]

Isolate	Year	ST	Voivodeship	City	Hospital	Number of SNPs	VIM variant	Remarks
4969-09[a]	2009	ST90	Śląskie	Katowice	HS777	0	VIM-4	ST90-In238-VIM-4; multiregional outbreak I
72-10	2010	ST90	Mazowieckie	Siedlce	HW222	19	VIM-4	ST90-In238-VIM-4; multiregional outbreak I
865-13	2013	ST90	Dolnośląskie	Wrocław	WD4	19	VIM-4	ST90-In238-VIM-4; multiregional outbreak I
5002-14	2014	ST90	Dolnośląskie	Legnica	HD1	20	VIM-4	ST90-In238-VIM-4; multiregional outbreak I
93-10	2010	ST90	Dolnośląskie	Legnica	HD1	21	VIM-4	ST90-In238-VIM-4; multiregional outbreak I
6580-16	2016	ST90	Śląskie	Katowice	HS3	22	VIM-4	ST90-In238-VIM-4; multiregional outbreak I
6004-12	2012	ST90	Wielkopolskie	Poznań	HP1	23	VIM-4	ST90-In238-VIM-4; multiregional outbreak I
1435-16	2016	ST90	Śląskie	Katowice	HS3	23	VIM-4	ST90-In238-VIM-4; multiregional outbreak I
3728-12	2012	ST90	Mazowieckie	Otwock	HWA5	28	VIM-4	ST90-In238-VIM-4; multiregional outbreak I
5917-12	2012	ST90	Świętokrzyskie	Skarżysko Kamienna	HT4	28	VIM-4	ST90-In238-VIM-4; multiregional outbreak I
9482-18	2018	ST90	Śląskie	Katowice	HS3	29	VIM-4	ST90-In238-VIM-4; multiregional outbreak I
9982-19	2019	ST90	Śląskie	Dąbrowa Górnicza	HS778	29	VIM-4	ST90-In238-VIM-4; multiregional outbreak I
6990-19	2019	ST90	Opolskie	Brzeg	HO3	32	VIM-4	ST90-In238-VIM-4; multiregional outbreak I
2015-10	2010	ST90	Dolnośląskie	Wrocław	HD3	33	VIM-4	ST90-In238-VIM-4; multiregional outbreak I
6360-12	2012	ST90	Mazowieckie	Radom	HM1	37	VIM-4	ST90-In238-VIM-4; multiregional outbreak I
3103-13	2013	ST90	Mazowieckie	Warszawa	HW11	37	VIM-4	ST90-In238-VIM-4; multiregional outbreak I
1847-19	2019	ST90	Świętokrzyskie	Końskie	HT7	38	VIM-4	ST90-In238-VIM-4; multiregional outbreak I
3529-19	2019	ST90	Świętokrzyskie	Końskie	HT7	38	VIM-4	ST90-In238-VIM-4; multiregional outbreak I
3601-19	2019	ST90	Świętokrzyskie	Końskie	HT7	38	VIM-4	ST90-In238-VIM-4; multiregional outbreak I
8732-11	2011	ST90	Dolnośląskie	Wrocław	HD3	39	VIM-4	ST90-In238-VIM-4; multiregional outbreak I
2144-16	2016	ST90	Wielkopolskie	Poznań	HP1	39	VIM-4	ST90-In238-VIM-4; multiregional outbreak I
6340-16	2016	ST90	Śląskie	Katowice	HS5	51	VIM-4	ST90-In238-VIM-4; multiregional outbreak I
4210-15	2015	ST90	Śląskie	Katowice	HS5	54	VIM-4	ST90-In238-VIM-4; multiregional outbreak I
4804-12	2012	ST90	Podkarpackie	Mielec	HR1	59	VIM-4	ST90-In238-VIM-4; multiregional outbreak I
723-15	2015	ST90	Małopolskie	Kraków	HK5	59	VIM-4	ST90-In238-VIM-4; multiregional outbreak I
6846-12	2012	ST90	Małopolskie	Kraków	HK5	60	VIM-4	ST90-In238-VIM-4; multiregional outbreak I
5242-12	2012	ST90	Małopolskie	Kraków	HK3	61	VIM-4	ST90-In238-VIM-4; multiregional outbreak I
5923-13	2013	ST90	Małopolskie	Kraków	HK3	61	VIM-4	ST90-In238-VIM-4; multiregional outbreak I
1399-10	2010	ST90	Wielkopolskie	Poznań	HP1	62	VIM-4	ST90-In238-VIM-4; multiregional outbreak I
6294-17	2017	ST90	Małopolskie	Kraków	HK5	62	VIM-4	ST90-In238-VIM-4; multiregional outbreak I
6293-09	2009	ST90	Podkarpackie	Mielec	HR1	63	VIM-4	ST90-In238a-VIM-4; multiregional outbreak I
6438-12	2012	ST90	Śląskie	Sosnowiec	AS1	63	VIM-4	ST90-In238-VIM-4; multiregional outbreak I
2794-19	2019	ST90	Małopolskie	Oświęcim	HK667	64	VIM-4	ST90-In238-VIM-4; multiregional outbreak I
2977-15	2015	ST90	Małopolskie	Kraków	HK5	65	VIM-4	ST90-In238-VIM-4; multiregional outbreak I
5884-19	2019	ST90	Śląskie	Katowice	HS26	65	VIM-4	ST90-In238-VIM-4; multiregional outbreak I
2224-10	2010	ST90	Wielkopolskie	Poznań	HP1	66	VIM-4	ST90-In238-VIM-4; multiregional outbreak I
478-12	2012	ST90	Mazowieckie	Warszawa	HW1	66	VIM-4	ST90-In238-VIM-4; multiregional outbreak I
2695-17	2017	ST90	Śląskie	Bystra	HS16	66	VIM-4	ST90-In238-VIM-4; multiregional outbreak I
6017-09	2009	ST90	Podkarpackie	Mielec	HR1	67	VIM-4	ST90-In238-VIM-4; multiregional outbreak I
6335-09	2009	ST90	Małopolskie	Kraków	HK6	67	VIM-4	ST90-In238-VIM-4; multiregional outbreak I
6229-10	2010	ST90	Podkarpackie	Rzeszów	HR888	68	VIM-4	ST90-In238-VIM-4; multiregional outbreak I
402-14	2014	ST90	Śląskie	Chorzów	HS2	68	VIM-4	ST90-In238-VIM-4; multiregional outbreak I
5237-14	2014	ST90	Lubelskie	Zamość	HL2	68	VIM-4	ST90-In238-VIM-4; multiregional outbreak I
5375-14	2014	ST90	Lubelskie	Zamość	HL2	68	VIM-4	ST90-In238-VIM-4; multiregional outbreak I
4034-10	2010	ST90	Lubuskie	Zielona Góra	HF2	68	VIM-4	ST90-In238-VIM-4; multiregional outbreak I
2072-14	2014	ST90	Śląskie	Sosnowiec	HS4	69	VIM-4	ST90-In238-VIM-4; multiregional outbreak I
216-15	2015	ST90	Lubelskie	Zamość	HL2	69	VIM-4	ST90-In238-VIM-4; multiregional outbreak I

Isolate	Year	ST	Voivodeship	City	Hospital	Number of SNPs	VIM variant	Remarks
9300-11	2011	ST90	Lubelskie	Lublin	HL444	70	VIM-4	ST90-In238-VIM-4; multiregional outbreak I
3571-13	2013	ST90	Małopolskie	Kraków	HK5	70	VIM-4	ST90-In238-VIM-4; multiregional outbreak I
3845-15	2015	ST90	Podkarpackie	Krosno	HR7	70	VIM-4	ST90-In238-VIM-4; multiregional outbreak I
5663-11	2011	ST90	Lubelskie	Zamość	HL2	71	VIM-4	ST90-In238-VIM-4; multiregional outbreak I
4158-13	2013	ST90	Podkarpackie	Jasło	HR4	71	VIM-4	ST90-In238-VIM-4; multiregional outbreak I
93-17	2017	ST90	Małopolskie	Kraków	HK8	71	VIM-4	ST90-In238-VIM-4; multiregional outbreak I
4761-18	2018	ST90	Lubelskie	Zamość	HL2	71	VIM-4	ST90-In238-VIM-4; multiregional outbreak I
9661-11	2011	ST90	Mazowieckie	Otwock	HWA3	72	VIM-4	ST90-In238-VIM-4; multiregional outbreak I
9960-11	2011	ST90	Podkarpackie	Mielec	HR1	72	VIM-4	ST90-In238-VIM-4; multiregional outbreak I
6442-12	2012	ST90	Podkarpackie	Łańcut	HR3	72	VIM-4	ST90-In238-VIM-4; multiregional outbreak I
4695-13	2013	ST90	Małopolskie	Kraków	HK5	72	VIM-4	ST90-In238-VIM-4; multiregional outbreak I
814-14	2014	ST90	Lubelskie	Lublin	HL5	72	VIM-4	ST90-In238-VIM-4; multiregional outbreak I
1152-14	2014	ST90	Lubelskie	Lublin	HL5	72	VIM-4	ST90-In238-VIM-4; multiregional outbreak I
1167-15	2015	ST90	Małopolskie	Limanowa	HK7	72	VIM-4	ST90-In238-VIM-4; multiregional outbreak I
584-17	2017	ST90	Świętokrzyskie	Starachowice	HT9	72	VIM-4	ST90-In238-VIM-4; multiregional outbreak I
7995-18	2018	ST90	Małopolskie	Kraków	HK16	72	VIM-4	ST90-In238-VIM-4; multiregional outbreak I
6234-09	2009	ST90	Podkarpackie	Mielec	HR1	73	VIM-4	ST90-In238-VIM-4; multiregional outbreak I
5310-10	2010	ST90	Podkarpackie	Rzeszów	HR888	73	VIM-4	ST90-In238a-VIM-4; multiregional outbreak I
9267-11	2011	ST90	Podkarpackie	Mielec	HR1	73	VIM-4	ST90-In238-VIM-4; multiregional outbreak I
447-13	2013	ST90	Lubelskie	Lublin	HL5	74	VIM-4	ST90-In238-VIM-4; multiregional outbreak I
2284-15	2015	ST90	Podkarpackie	Łańcut	HR3	74	VIM-4	ST90-In238-VIM-4; multiregional outbreak I
4591-15	2015	ST90	Pomorskie	Gdańsk	HG1	74	VIM-4	ST90-In238-VIM-4; multiregional outbreak I
3356-16	2016	ST90	Małopolskie	Kraków	HK5	74	VIM-4	ST90-In238-VIM-4; multiregional outbreak I
2521-16	2016	ST90	Dolnośląskie	Polanica-Zdrój	HD11	74	VIM-4	ST90-In238-VIM-4; multiregional outbreak I
444-11	2011	ST90	Podkarpackie	Mielec	HR1	75	VIM-4	ST90-In238-VIM-4; multiregional outbreak I
1900-14	2014	ST90	Lubelskie	Puławy	HL6	75	VIM-4	ST90-In238-VIM-4; multiregional outbreak I
875-17	2017	ST90	Małopolskie	Kraków	HK5	75	VIM-4	ST90-In238-VIM-4; multiregional outbreak I
7625-18	2018	ST90	Podkarpackie	Jasło	HR4	76	VIM-4	ST90-In238-VIM-4; multiregional outbreak I
4738-15	2015	ST90	Podkarpackie	Łańcut	HR3	77	VIM-4	ST90-In238-VIM-4; multiregional outbreak I
7818-17	2017	ST90	Małopolskie	Limanowa	HK7	77	VIM-4	ST90-In238-VIM-4; multiregional outbreak I
2218-14	2014	ST90	Podkarpackie	Jasło	AR2	78	VIM-4	ST90-In238-VIM-4; multiregional outbreak I
218-17	2017	ST90	Podkarpackie	Rzeszów	HR5	78	VIM-4	ST90-In238-VIM-4; multiregional outbreak I
5399-17	2017	ST90	Dolnośląskie	Wrocław	HD7	78	VIM-4	ST90-In238a-VIM-4; multiregional outbreak I
7398-17	2017	ST90	Małopolskie	Limanowa	HK7	78	VIM-4	ST90-In238-VIM-4; multiregional outbreak I
7344-18	2018	ST90	Podkarpackie	Jasło	HR4	79	VIM-4	ST90-In238-VIM-4; multiregional outbreak I
4148-13	2013	ST90	Śląskie	Chorzów	HS2	80	VIM-4	ST90-In238-VIM-4; multiregional outbreak I
4149-13	2013	ST90	Śląskie	Chorzów	HS2	80	VIM-4	ST90-In238-VIM-4; multiregional outbreak I
41-17	2016	ST90	Dolnośląskie	Polanica-Zdrój	HD11	80	VIM-4	ST90-In238-VIM-4; multiregional outbreak I
4410-13	2013	ST90	Podkarpackie	Jasło	HR4	81	VIM-4	ST90-In238-VIM-4; multiregional outbreak I
4537-13	2013	ST90	Śląskie	Chorzów	HS2	81	VIM-4	ST90-In238-VIM-4; multiregional outbreak I
1000-11	2011	ST90	Podkarpackie	Mielec	HR1	82	VIM-4	ST90-In238-VIM-4; multiregional outbreak I
4244-18	2018	ST90	Małopolskie	Andrychów	AK3	82	VIM-4	ST90-In238-VIM-4; multiregional outbreak I
825-14	2014	ST90	Podkarpackie	Jasło	HR4	83	VIM-4	ST90-In238-VIM-4; multiregional outbreak I
4928-11	2011	ST90	Małopolskie	Kraków	HK2	84	VIM-4	ST90-In238-VIM-4; multiregional outbreak I
5072-10	2010	ST90	Świętokrzyskie	Kielce	HT555	85	VIM-4	ST90-In238-VIM-4; multiregional outbreak I
5808-18	2018	ST90	Podkarpackie	Mielec	HR1	85	VIM-4	ST90-In238-VIM-4; multiregional outbreak I
7381-18	2018	ST90	Pomorskie	Wejherowo	HG7	85	VIM-4	ST90-In238-VIM-4; multiregional outbreak I
7205-19	2019	ST90	Podkarpackie	Tarnobrzeg	HR889	85	VIM-4	ST90-In238-VIM-4; multiregional outbreak I

Isolate	Year	ST	Voivodeship	City	Hospital	Number of SNPs	VIM variant	Remarks
3337-14	2014	ST90	Podkarpackie	Jasło	HR4	86	VIM-4	ST90-In238-VIM-4; multiregional outbreak I
144-14	2013	ST90	Małopolskie	Kraków	HK2	89	VIM-4	ST90-In238-VIM-4; multiregional outbreak I
3253-13	2013	ST90	Podkarpackie	Rzeszów	HR5	90	VIM-4	ST90-In238a-VIM-4; multiregional outbreak I
6747-18	2018	ST90	Lubelskie	Lublin	HL11	91	VIM-4	ST90-In238-VIM-4; multiregional outbreak I
6074-17	2017	ST90	Podkarpackie	Krosno	HR7	92	VIM-4	ST90-In238-VIM-4; multiregional outbreak I
6754-17	2017	ST90	Podkarpackie	Przemyśl	HR11	92	VIM-4	ST90-In238a-VIM-4; multiregional outbreak I
8600-19	2019	ST90	Śląskie	Siemianowice Śl.	HS1	93	VIM-4	ST90-In238-VIM-4; multiregional outbreak I
6492-18	2018	ST90	Małopolskie	Kraków	HK8	96	VIM-4	ST90-In238-VIM-4; multiregional outbreak I
6039-19	2019	ST90	Małopolskie	Kraków	HK5	115	VIM-4	ST90-In238-VIM-4; multiregional outbreak I
3935-16	2016	ST90	Podkarpackie	Mielec	HR1	116	VIM-4	ST90-In238-VIM-4; multiregional outbreak I
7910-19	2019	ST1762	Podkarpackie	Tarnobrzeg	HR890	127	VIM-4	ST1762-In238-VIM-4; multiregional outbreak I
3238-16	2016	ST1762	Podkarpackie	Nowa Dęba	HR8	132	VIM-4	ST1762-In238-VIM-4; multiregional outbreak I
5178-18	2018	ST90	Lubelskie	Lublin	HL14	133	VIM-4	ST90-In238-VIM-4; multiregional outbreak I
6501-12	2012	ST90	Małopolskie	Bochnia	HK4	135	VIM-4	ST90-In238-VIM-4; multiregional outbreak I
3526-14	2014	ST90	Mazowieckie	Radom	HM11	159	VIM-4	ST90-In238-VIM-4; multiregional outbreak I
9518-19	2019	ST90	Małopolskie	Kraków	HK5	170	VIM-4	ST90-In238-VIM-4; multiregional outbreak I
3525-14	2014	ST90	Mazowieckie	Radom	HM11	192	VIM-4	ST90-In238-VIM-4; multiregional outbreak I
3524-14	2014	ST90	Mazowieckie	Radom	HM11	207	VIM-4	ST90-In238-VIM-4; multiregional outbreak I
10235-19	2019	ST90	Śląskie	Chorzów	HS2	649	VIM-1	ST90-In237a-VIM-1; single case
4083-09	2009	ST90	Mazowieckie	Warszawa	HW4	1631	VIM-4	ST90-In238-VIM-4; single case
3041-15	2015	ST90	Dolnośląskie	Wrocław	WD7	1653	VIM-1	ST90-In916-VIM-1; single case
2091-19	2019	ST90	Łódzkie	Łódź	HE7	1665	VIM-1	ST90-In916-VIM-1; single case
7154-17	2017	ST90	Dolnośląskie	Wrocław	WD7	1669	VIM-4+IMP-19	ST90-In238a-VIM-4-In2241-IMP-19; hospital dissemination
3723-18	2018	ST90	Dolnośląskie	Wrocław	WD7	1670	VIM-4+IMP-19	ST90-In238a-VIM-4-In2241-IMP-19; hospital dissemination

[a] – reference isolate, *i.e.* the Poland's index isolate of ST90/CC90 as confirmed by the National Reference Centre for Susceptibility Testing
[b] – the SNP analysis of the CC90 isolates revealed 4092 polymorphic positions within ~4.0 Mb (77%) of the reference genome.

Appendix Table 5. SNP scores between *E. hormaechei* subs. *steigerwaltii* ST89 isolates[a,b]

Isolate	Year	Voivodeship	City	Hospital	Number of SNPs	VIM variant	Remarks
2944-06[a]	2006	Lubuskie	Nowa Sól	HF20	0	VIM-20	ST89-In1444-VIM-20 regional outbreak III
5014-10	2010	Wielkopolskie	Poznań	HP2	28	VIM-20	ST89-In1444-VIM-20 regional outbreak III
258-11	2011	Wielkopolskie	Poznań	HP2	28	VIM-20	ST89-In1444-VIM-20 regional outbreak III
3033-13	2013	Wielkopolskie	Konin	HP7	28	VIM-20	ST89-In1444-VIM-20 regional outbreak III
5474-10	2010	Wielkopolskie	Poznań	HP2	30	VIM-20	ST89-In1444-VIM-20 regional outbreak III
9056-11	2011	Wielkopolskie	Poznań	HP100	30	VIM-20	ST89-In1444-VIM-20 regional outbreak III
2543-14	2014	Wielkopolskie	Gniezno	HP14	32	VIM-20	ST89-In1444-VIM-20 regional outbreak III
274-15	2015	Wielkopolskie	Piła	HP17	35	VIM-20	ST89-In1444-VIM-20 regional outbreak III
6630-12	2012	Wielkopolskie	Poznań	HP1	38	VIM-20	ST89-In1444-VIM-20 regional outbreak III
1064-16	2016	Wielkopolskie	Poznań	HP9	48	VIM-20	ST89-In1444-VIM-20 regional outbreak III
3584-16	2016	Lubuskie	Zielona Góra	AF1	49	VIM-20	ST89-In1444-VIM-20 regional outbreak III
195-11	2011	Mazowieckie	Grodzisk Mazowiecki	HW100	32	VIM-20	ST89-In1444-VIM-20 regional outbreak III
4884-09	2009	Dolnośląskie	Wrocław	HD100	56	VIM-2	ST89-In1008-VIM-2 hospital outbreak
4885-09	2009	Dolnośląskie	Wrocław	HD100	56	VIM-2	ST89-In1008-VIM-2 hospital outbreak
8770-11	2011	Kujawsko-Pomorskie	Bydgoszcz	HC1	73	VIM-40	ST89-In1445-VIM-40 regional outbreak IV
5715-12	2012	Kujawsko-Pomorskie	Bydgoszcz	HC1	77	VIM-40	ST89-In1445-VIM-40 regional outbreak IV
3744-12	2012	Kujawsko-Pomorskie	Inowrocław	HC3	78	VIM-40	ST89-In1445-VIM-40 regional outbreak IV
533-12	2012	Kujawsko-Pomorskie	Bydgoszcz	HC1	80	VIM-40	ST89-In1445-VIM-40 regional outbreak IV
4261-12	2012	Kujawsko-Pomorskie	Inowrocław	HC3	80	VIM-40	ST89-In1445-VIM-40 regional outbreak IV
5863-12	2012	Kujawsko-Pomorskie	Inowrocław	HC3	85	VIM-40	ST89-In1445-VIM-40 regional outbreak IV
479-12	2012	Mazowieckie	Warszawa	HW9	77	VIM-40	ST89-In1445-VIM-40 regional outbreak IV
4642-14	2014	Mazowieckie	Mińsk Mazowiecki	HM2	52	VIM-4	ST89-In238-VIM-4 single case
43-16	2015	Mazowieckie	Siedlce	HM10	92	VIM-4	ST89-In2238-VIM-4 single case
1071-16	2016	Podlaskie	Białystok	HB20	110	VIM-4	ST89-In238-VIM-4 single case
1100-16	2016	Lubuskie	Gorzów Wielkopolski	HF5	122	VIM-4	ST89-In238-VIM-4 single case
7753-18	2018	Łódzkie	Łódź	HE7	117	VIM-1	ST89-In916-VIM-1 regional outbreak II
9792-19	2019	Łódzkie	Łódź	HE7	117	VIM-1	ST89-In916-VIM-1 regional outbreak II
7654-19	2019	Łódzkie	Łódź	HE7	118	VIM-1	ST89-In916-VIM-1 regional outbreak II
7780-19	2019	Łódzkie	Łódź	HE112	119	VIM-1	ST89-In916-VIM-1 regional outbreak II
9398-18	2018	Łódzkie	Łódź	HE7	119	VIM-1	ST89-In916-VIM-1 regional outbreak II
6188-18	2018	Łódzkie	Łódź	HE7	120	VIM-1	ST89-In916-VIM-1 regional outbreak II
8201-18	2018	Łódzkie	Łódź	HE7	120	VIM-1	ST89-In916-VIM-1 regional outbreak II
3578-16	2016	Łódzkie	Łódź	HE7	126	VIM-1	ST89-In916-VIM-1 regional outbreak II
7339-17	2017	Łódzkie	Łódź	HE7	127	VIM-1	ST89-In916-VIM-1 regional outbreak II
8339-17	2017	Łódzkie	Łódź	HE7	127	VIM-1	ST89-In916-VIM-1 regional outbreak II
4253-16	2016	Łódzkie	Łódź	HE7	128	VIM-1	ST89-In916-VIM-1 regional outbreak II
4988-16	2016	Łódzkie	Łódź	HE7	129	VIM-1	ST89-In916-VIM-1 regional outbreak II
2917-18	2018	Łódzkie	Łódź	HE7	130	VIM-1	ST89-In916-VIM-1 regional outbreak II
7815-17	2017	Łódzkie	Łódź	HE7	130	VIM-1	ST89-In916-VIM-1 regional outbreak II
2284-18	2018	Łódzkie	Łódź	HE7	131	VIM-1	ST89-In916-VIM-1 regional outbreak II
6087-18	2018	Łódzkie	Łódź	HE111	131	VIM-1	ST89-In916-VIM-1 regional outbreak II
7133-16	2016	Łódzkie	Łódź	HE7	132	VIM-1	ST89-In916-VIM-1 regional outbreak II
7382-16	2016	Łódzkie	Łódź	HE7	132	VIM-1	ST89-In916-VIM-1 regional outbreak II
7338-17	2017	Łódzkie	Łódź	HE7	132	VIM-1	ST89-In916-VIM-1 regional outbreak II
5796-16	2016	Łódzkie	Łódź	HE7	133	VIM-1	ST89-In916-VIM-1 regional outbreak II
7019-16	2016	Łódzkie	Łódź	HE7	133	VIM-1	ST89-In916-VIM-1 regional outbreak II
1686-17	2017	Łódzkie	Łódź	HE7	133	VIM-1	ST89-In916-VIM-1 regional outbreak II

Isolate	Year	Voivodeship	City	Hospital	Number of SNPs	VIM variant	Remarks
7020-16	2016	Łódzkie	Łódź	HE7	134	VIM-1	ST89-In916-VIM-1 regional outbreak II
7383-16	2016	Łódzkie	Łódź	HE7	135	VIM-1	ST89-In916-VIM-1 regional outbreak II
4051-17	2017	Łódzkie	Łódź	HE17	135	VIM-1	ST89-In916-VIM-1 regional outbreak II
7517-19	2019	Łódzkie	Łódź	HE13	135	VIM-1	ST89-In916-VIM-1 regional outbreak II
6901-17	2017	Łódzkie	Łódź	HE7	136	VIM-1	ST89-In916-VIM-1 regional outbreak II
7381-16	2016	Łódzkie	Łódź	HE7	138	VIM-1	ST89-In916-VIM-1 regional outbreak II
1175-17	2017	Łódzkie	Łódź	HE7	138	VIM-1	ST89-In916-VIM-1 regional outbreak II
357-19	2019	Łódzkie	Łódź	HE7	140	VIM-1	ST89-In916-VIM-1 regional outbreak II
775-19	2019	Łódzkie	Łódź	HE7	140	VIM-1	ST89-In916-VIM-1 regional outbreak II
938-19	2019	Łódzkie	Łódź	HE7	140	VIM-1	ST89-In916-VIM-1 regional outbreak II
1008-19	2019	Łódzkie	Łódź	HE7	140	VIM-1	ST89-In916-VIM-1 regional outbreak II
1118-19	2019	Łódzkie	Łódź	HE7	140	VIM-1	ST89-In916-VIM-1 regional outbreak II
1888-19	2019	Łódzkie	Łódź	HE7	141	VIM-1	ST89-In916-VIM-1 regional outbreak II
7813-17	2017	Łódzkie	Łódź	HE7	141	VIM-1	ST89-In916-VIM-1 regional outbreak II
5105-19	2019	Łódzkie	Łódź	HE7	142	VIM-1	ST89-In916-VIM-1 regional outbreak II
5431-19	2019	Łódzkie	Łódź	HE7	142	VIM-1	ST89-In916-VIM-1 regional outbreak II
5741-19	2019	Łódzkie	Łódź	HE7	142	VIM-1	ST89-In916-VIM-1 regional outbreak II
9991-19	2019	Łódzkie	Łódź	HE7	142	VIM-1	ST89-In916-VIM-1 regional outbreak II
9312-19	2019	Łódzkie	Łódź	HE7	145	VIM-1	ST89-In916-VIM-1 regional outbreak II
9794-19	2019	Łódzkie	Łódź	HE7	150	VIM-1	ST89-In916-VIM-1 regional outbreak II
10251-19	2019	Łódzkie	Łódź	HE7	150	VIM-1	ST89-In916-VIM-1 regional outbreak II
8253-19	2019	Łódzkie	Radomsko	HE20	137	VIM-4	ST89-In1654-VIM-4 single case
6995-19	2019	Wielkopolskie	Poznań	HP11	131	VIM-1	ST89-In916-VIM-1 regional outbreak II
7480-19	2019	Wielkopolskie	Poznań	HP11	131	VIM-1	ST89-In916-VIM-1 regional outbreak II
5973-16	2016	Wielkopolskie	Poznań	HP11	132	VIM-1	ST89-In916-VIM-1 regional outbreak II
6126-18	2018	Wielkopolskie	Poznań	HP11	132	VIM-1	ST89-In916-VIM-1 regional outbreak II
5179-18	2018	Pomorskie	Wejherowo	HG7	122	VIM-1	ST89-In916-VIM-1 regional outbreak II

[a] – reference isolate, *i.e.* the Poland's index isolate of ST89 as confirmed by the National Reference Centre for Susceptibility Testing
[b] – the SNP analysis of the ST89 isolates revealed 797 polymorphic positions within ~4.3 Mb (87%) of the reference genome.

Appendix Table 6. SNP scores between *E. hormaechei* subs. *xiangfangensis* CC121 (ST121 and ST1756) isolates[a,b]

Isolate	Year	ST	Voivodeship	City	Hospital	Number of SNPs	VIM variant	Remarks
743-14[a]	2014	ST121	Mazowieckie	Warszawa	HW1	0	VIM-1	ST121-In916-VIM-1 interregional outbreak V
2876-15	2015	ST121	Mazowieckie	Warszawa	HW1	9	VIM-1	ST121-In916-VIM-1 interregional outbreak V
2902-15	2015	ST121	Mazowieckie	Otwock	HWA5	10	VIM-1	ST121-In916-VIM-1 interregional outbreak V
720-19	2019	ST121	Mazowieckie	Warszawa	HW9	30	VIM-1	ST121-In916-VIM-1 interregional outbreak V
4979-19	2019	ST121	Mazowieckie	Płońsk	HM4	30	VIM-1	ST121-In916-VIM-1 interregional outbreak V
2674-19	2019	ST121	Mazowieckie	Płońsk	HM4	31	VIM-1	ST121-In916-VIM-1 interregional outbreak V
5458-19	2019	ST121	Warmińsko-Mazurskie	Węgorzewo	HN9	31	VIM-1	ST121-In916-VIM-1 interregional outbreak V
6697-19	2019	ST121	Mazowieckie	Płońsk	HM4	31	VIM-1	ST121-In916-VIM-1 interregional outbreak V
7955-19	2019	ST121	Mazowieckie	Płońsk	HM4	31	VIM-1	ST121-In916-VIM-1 interregional outbreak V
3546-14	2014	ST121	Mazowieckie	Warszawa	HW1	34	VIM-1	ST121-In916-VIM-1 interregional outbreak V
2132-19	2019	ST121	Mazowieckie	Płońsk	HM4	36	VIM-1	ST121-In916-VIM-1 interregional outbreak V
188-16	2015	ST121	Mazowieckie	Warszawa	HW1	37	VIM-1	ST121-In916-VIM-1 interregional outbreak V
4600-15	2015	ST121	Mazowieckie	Warszawa	HW1	38	VIM-1	ST121-In916-VIM-1 interregional outbreak V
4220-14	2014	ST121	Łódzkie	Łódź	HE2	43	VIM-1	ST121-In916-VIM-1 interregional outbreak V
4728-14	2014	ST121	Łódzkie	Łódź	HE2	43	VIM-1	ST121-In916-VIM-1 interregional outbreak V
2977-14	2014	ST121	Dolnośląskie	Wrocław	HD999	44	VIM-1	ST121-In916-VIM-1 interregional outbreak V
4221-14	2014	ST121	Łódzkie	Łódź	HE2	44	VIM-1	ST121-In916-VIM-1 interregional outbreak V
8378-18	2018	ST121	Łódzkie	Łódź	HE7	50	VIM-1	ST121-In916-VIM-1 interregional outbreak V
209-18	2017	ST121	Podkarpackie	Przemyśl	HR11	56	VIM-1	ST121-In916-VIM-1 interregional outbreak V
7752-18	2018	ST1756	Łódzkie	Łódź	HE7	57	VIM-1	ST1756-In916-VIM-1 interregional outbreak V
4480-19	2019	ST1756	Łódzkie	Łódź	HE6	59	VIM-1	ST1756-In916-VIM-1 interregional outbreak V
1612-18	2018	ST121	Podkarpackie	Przemyśl	HR11	59	VIM-1	ST121-In916-VIM-1 interregional outbreak V
6350-18	2018	ST121	Podkarpackie	Przemyśl	HR11	64	VIM-1	ST121-In916-VIM-1 interregional outbreak V
5793-19	2019	ST1756	Łódzkie	Łódź	HE7	67	VIM-1	ST1756-In916-VIM-1 interregional outbreak V
7328-19	2019	ST121	Dolnośląskie	Wrocław	HD7	68	VIM-1	ST121-In916-VIM-1 interregional outbreak V
8602-19	2019	ST121	Podkarpackie	Rzeszów	HR17	68	VIM-1	ST121-In916-VIM-1 interregional outbreak V
5975-19	2019	ST121	Wielkopolskie	Ostrów Wielkopolski	HP18	70	VIM-1	ST121-In916-VIM-1 interregional outbreak V
7573-18	2018	ST121	Mazowieckie	Otwock	HWA2	34	VIM-4	ST121-In238a-VIM-4 regional outbreak VI
2589-19	2019	ST121	Mazowieckie	Otwock	HWA1	35	VIM-4	ST121-In238a-VIM-4 regional outbreak VI
3905-19	2019	ST121	Mazowieckie	Otwock	HWA1	37	VIM-4	ST121-In238a-VIM-4 regional outbreak VI
4784-19	2019	ST121	Mazowieckie	Warszawa	HW22	38	VIM-4	ST121-In238a-VIM-4 regional outbreak VI
5085-19	2019	ST121	Mazowieckie	Otwock	HWA3	40	VIM-4	ST121-In238a-VIM-4 regional outbreak VI
5713-17	2017	ST121	Mazowieckie	Warszawa	HW22	41	VIM-4	ST121-In238a-VIM-4 regional outbreak VI
7311-17	2017	ST121	Mazowieckie	Warszawa	HW21	70	VIM-2	ST121-In2242-VIM-2 regional dissemination
1969-19	2019	ST121	Mazowieckie	Konstancin-Jeziorna	HWA9	71	VIM-2	ST121-In2242-VIM-2 regional dissemination
1966-19	2019	ST121	Mazowieckie	Grójec	HM13	35	VIM-4	ST121-In238-VIM-4 single case
9390-18	2018	ST121	Mazowieckie	Warszawa	HW6	45	VIM-4	ST121-In238-VIM-4 single case
883-16	2016	ST121	Lubuskie	Gorzów Wielkopolski	HF5	75	VIM-4	ST121-In238-VIM-4 single case
6808-19	2019	ST121	Mazowieckie	Warszawa	HW567	84	VIM-4	ST121-In2016a-VIM-4 single case

[a] – reference isolate, *i.e.* the Poland's first index isolate of ST121/CC121 as confirmed by the National Reference Centre for Susceptibility Testing
[b] – the SNP analysis of the CC121 isolates revealed 559 polymorphic positions within ~4.6 Mb (85%) of the reference genome.

Appendix Table 7. SNP scores between *E. hormaechei* subs. *xiangfangensis* CG66 (ST66 and ST1754) isolates[a,b]

Isolate	Year	ST	Voivodeship	City	Hospital	Number of SNPs	VIM variant	Remarks
5955-16[a]	2016	ST66	Mazowieckie	Majdan	HWA16	0	VIM-1	ST66-In916-VIM-1 interregional outbreak VII
1881-18	2018	ST66	Łódzkie	Łódź	HE13	10	VIM-1	ST66-In916-VIM-1 interregional outbreak VII
6334-17	2017	ST66	Łódzkie	Piotrków Trybunalski	HE19	13	VIM-1	ST66-In916-VIM-1 interregional outbreak VII
8063-17	2017	ST66	Łódzkie	Piotrków Trybunalski	HE19	13	VIM-1	ST66-In916-VIM-1 interregional outbreak VII
5530-19	2019	ST66	Mazowieckie	Majdan	HWA16	13	VIM-1	ST66-In916-VIM-1 interregional outbreak VII
2265-18	2018	ST66	Łódzkie	Zgierz	HE8	17	VIM-1	ST66-In916-VIM-1 interregional outbreak VII
1511-18	2018	ST66	Łódzkie	Łódź	HE11	18	VIM-1	ST66-In916-VIM-1 interregional outbreak VII
5642-17	2017	ST66	Łódzkie	Łódź	HE10	19	VIM-1	ST66-In916-VIM-1 interregional outbreak VII
5769-17	2017	ST66	Dolnośląskie	Wrocław	HD7	404	VIM-1	ST66-In916-VIM-1 regional outbreak VIII
7153-17	2017	ST66	Dolnośląskie	Wrocław	HD7	406	VIM-1	ST66-In916-VIM-1 regional outbreak VIII
7635-17	2017	ST66	Dolnośląskie	Wrocław	HD7	407	VIM-1	ST66-In916-VIM-1 regional outbreak VIII
5771-17	2017	ST66	Dolnośląskie	Wrocław	HD7	409	VIM-1	ST66-In916-VIM-1 regional outbreak VIII
6937-17	2017	ST66	Dolnośląskie	Wrocław	HD7	410	VIM-1	ST66-In916-VIM-1 regional outbreak VIII
5891-18	2018	ST66	Dolnośląskie	Wrocław	HD7	412	VIM-1	ST66-In916-VIM-1 regional outbreak VIII
9014-17	2017	ST66	Dolnośląskie	Wrocław	HD7	413	VIM-1	ST66-In916-VIM-1 regional outbreak VIII
9015-17	2017	ST66	Dolnośląskie	Wrocław	HD7	413	VIM-1	ST66-In916-VIM-1 regional outbreak VIII
6621-17	2017	ST66	Dolnośląskie	Wrocław	HD7	417	VIM-1	ST66-In916-VIM-1 regional outbreak VIII
6434-18	2018	ST1754	Dolnośląskie	Wrocław	HD101	425	VIM-1	ST1754-In916-VIM-1 regional outbreak VIII
6779-19	2019	ST66	Dolnośląskie	Wrocław	HD19	431	VIM-1	ST66-In916-VIM-1 regional outbreak VIII

[a] – reference isolate, *i.e.* the Poland's index isolate of ST66/CG66 as confirmed by the National Reference Centre for Susceptibility Testing
[b] – the SNP analysis of the CG66 isolates revealed 511 polymorphic positions within ~4.5 Mb (88%) of the reference genome.

Appendix Table 8. SNP scores between *E. hormaechei* subs. *steigerwaltii* ST134 isolates[a,b]

Isolate	Year	Voivodeship	City	Hospital	Number of SNPs	VIM variant	Remarks
5435-13[a]	2013	Mazowieckie	Warszawa	HW1	0	VIM-4	ST134-In238-VIM-4 regional outbreak IX
5436-13	2013	Mazowieckie	Warszawa	HW1	7	VIM-4	ST134-In238-VIM-4 regional outbreak IX
2118-14	2014	Mazowieckie	Warszawa	HW1	14	VIM-4	ST134-In238-VIM-4 regional outbreak IX
1958-15	2015	Mazowieckie	Warszawa	HW9	25	VIM-4	ST134-In238-VIM-4 regional outbreak IX
6302-16	2016	Mazowieckie	Warszawa	HW24	29	VIM-4	ST134-In238-VIM-4 regional outbreak IX
5897-16	2016	Mazowieckie	Warszawa	HW6	30	VIM-4	ST134-In238-VIM-4 regional outbreak IX
1258-16	2016	Mazowieckie	Warszawa	HW24	30	VIM-4	ST134-In238-VIM-4 regional outbreak IX
885-16	2016	Mazowieckie	Warszawa	HW24	31	VIM-4	ST134-In238-VIM-4 regional outbreak IX
151-17	2017	Mazowieckie	Warszawa	HW24	31	VIM-4	ST134-In238-VIM-4 regional outbreak IX
2791-19	2019	Świętokrzyskie	Kielce	HT6	192	VIM-1	ST134-In916-VIM-1 hospital dissemination
3340-19	2019	Świętokrzyskie	Kielce	HT6	193	VIM-1	ST134-In916-VIM-1 hospital dissemination
4986-14	2014	Dolnośląskie	Wrocław	HD4	208	VIM-4	ST134-In238a-VIM-4 hospital dissemination
752-15	2015	Dolnośląskie	Wrocław	HD4	211	VIM-4	ST134-In238a-VIM-4 hospital dissemination

[a] – reference isolate, *i.e.* the Poland's index isolate of ST134 as confirmed by the National Reference Centre for Susceptibility Testing
[b] – the SNP analysis of the ST134 isolates revealed 307 polymorphic positions within ~4.5 Mb (89%) of the reference genome.

Appendix Table 9. Resistomes of the *Enterobacter* spp. isolates

taxa/ST	integron	n isolates	remarks	acquired AMR genes[a]: resistance to β-lactams	acquired AMR genes[a]: resistance to aminoglycosides	acquired AMR genes[a]: resistance to other groups	n AMR genes/ isolate
E. hormaechei subsp. *steigerwaltii*							
ST45	In238	7		*bla*$_{\text{VIM-4}}$, (*bla*$_{\text{CTX-M-3}}$), (*bla*$_{\text{CMY-83}}$), *bla*$_{\text{SHV-5}}$	(*aac(3)-Ia*), (*aac(6')-Im*), *aac(6')-Ib*, (*aadA1*), (*aph(2'')-IIa*), (*aph(3'')-Ib*), *aph(6)-Id*	(*dfrA1*), *dfrA14*, *sul1*, (*tet(A)*), *tet(B)*, (*catA1*), (*catA2*), *oqxB*	12.3
	In916	2		*bla*$_{\text{VIM-1}}$, (*bla*$_{\text{CTX-M-9}}$), (*bla*$_{\text{SHV-12}}$), *bla*$_{\text{TEM-1}}$	*aac(6')-Ib*, *aadA1*, (*aadA2*), (*ant(2'')-Ia*), *aph(3'')-Ib*, *aph(6)-Id*, *aph(3')-XV*	*dfrA14*, *sul1*, *sul2*, (*qnrA1*), (*qnrS1*), (*tet(A)*), *catA2*, *catB2*, *oqxB*, (*mcr-9.1*)	18.0
ST62	In916	1		*bla*$_{\text{VIM-1}}$, *bla*$_{\text{SHV-12}}$, *bla*$_{\text{LAP-2}}$	*aac(6')-Ib*, *aadA1*, *aadA16*, *aph(3'')-Ib*, *aph(6)-Id*, *aph(3')-XV*	*dfrA14*, *dfrA27*, *sul1*, *sul2*, *qnrS1*, *fosA*, *tet(A)*, *catB2*, *oqxB*, *arr*	19.0
ST89	In916	48	outbreak II	*bla*$_{\text{VIM-1}}$, (*bla*$_{\text{CTX-M-3}}$), (*bla*$_{\text{CTX-M-15}}$), (***bla*$_{\text{CTX-M-256}}$**),[b] (*bla*$_{\text{SHV-12}}$), (*bla*$_{\text{OXA-1}}$), (*bla*$_{\text{TEM-1}}$)	(*aac(3)-IId*), (*aac(3)-IIe*), *aac(6')-Ib*, (*aac(6')-Ib-cr*), *aadA1*, (*aph(3'')-Ib*), (*aph(6)-Id*), *aph(3')-XV*	(*sat2*), (*dfrA1*), (*dfrA14*), (*sul1*), (*sul2*), (*qnrS1*), *fosA*, (*tet(A)*), (*catA1*), *catB2*, (*catB3*), (*oqxA*), (*oqxB*), (*mphA*)	19.6
	In238	3		*bla*$_{\text{VIM-4}}$, (*bla*$_{\text{CTX-M-3}}$), (*bla*$_{\text{SHV-12}}$), (*bla*$_{\text{TEM-1}}$)	(*aac(3)-IIe*), *aac(6')-Ib*, (*aadA2*), (*aph(3')-VIa*), (*aph(3'')-Ib*), (*aph(6)-Id*), *armA*	(*sat2*), (*dfrA1*), (*dfrA12*), (*dfrA19*), (*sul1*), (*sul2*), *fosA*, (*tet(D)*), *mphE*, *msrE*, *oqxB*, (*mcr-9.1*)	14.7
	In1654	1		*bla*$_{\text{VIM-4}}$, *bla*$_{\text{CTX-M-3}}$, *bla*$_{\text{OXA-1}}$	*aadA2*, *ant(2'')-Ia*, *armA*	*sat2*, *dfrA1*, *dfrA12*, *sul1*, *qnrS1*, *fosA*, *tet(A)*, *mphE*, *msrE*	15.0
	**In2238**	1		*bla*$_{\text{VIM-4}}$, *bla*$_{\text{CTX-M-3}}$, *bla*$_{\text{OXA-2}}$, *bla*$_{\text{TEM-1}}$	*aac(6')-Ib*, *aadA1*, *aadA2*, *armA*	*dfrA1*, *dfrA12*, *sul1*, *fosA*, *catA1*, *msrE*, *oqxB*	15.0
	In1445	7	outbreak IV	*bla*$_{\text{VIM-40}}$, *bla*$_{\text{CTX-M-3}}$	*aac(6')-Ib*, (*aph(3')-VI*), *aph(3'')-Ib*, *aph(6)-Id*	*sul1*, *fosA*, *oqxB*	9.8
	In1008	2		*bla*$_{\text{VIM-2}}$, *bla*$_{\text{CTX-M-3}}$, *bla*$_{\text{SHV-5}}$	*aac(6')-Ib*, *aadA2*, *armA*	*dfrA12*, (*sul1*), *fosA*, (*tet(A)*), *mphE*, *msrE*, (*oqxB*)	12.5
	In1444	12	outbreak III	*bla*$_{\text{VIM-20}}$, (*bla*$_{\text{CTX-M-3}}$)	(*aac(3)-IId*), *aac(6')-Ib*, (*aadA2*), (*aph(3')-Ia*), (*armA*)	(*dfrA12*), *sul1*, *fosA*, (*mphE*), (*msrE*), *oqxB*	12.6
ST90	In238	107	outbreak I	*bla*$_{\text{VIM-4}}$, (*bla*$_{\text{CTX-M-3}}$), (*bla*$_{\text{CTX-M-15}}$), (*bla*$_{\text{DHA-1}}$), (*bla*$_{\text{SHV-5}}$), (*bla*$_{\text{SHV-12}}$), (*bla*$_{\text{OXA-1}}$), (*bla*$_{\text{OXA-10}}$), (*bla*$_{\text{TEM-1}}$), (*bla*$_{\text{LAP-2}}$)	(*aac(3)-IIa*), (*aac(3)-IId*), (*aac(3)-IIe*), (*aac(6')-IIc*), *aac(6')-Ib*, (*aac(6')-Ib-cr*), (*aadA1*), (*aadA2*), (*ant(2'')-Ia*), (*aph(2'')-IIa*), (*aph(3')-Ia*), (*aph(3'')-Ib*), (*aph(6)-Id*), (*armA*)	(*dfrA1*), (*dfrA12*), (*dfrA14*), (*dfrA19*), (*sul1*), (*sul2*), (*qnrA1*), (*qnrB1*), (*qnrB4*), (*qnrS1*), (*fosA*), (*tet(A)*), (*tet(D)*), (*catA2*), (*catB3*), (*cmlA1*), (*mphE*), (*msrE*), (*oqxB*), (*mcr-9.1*), (*arr*)	14.6
	In238a	5		*bla*$_{\text{VIM-4}}$, *bla*$_{\text{CTX-M-3}}$, (*bla*$_{\text{SHV-12}}$), (*bla*$_{\text{TEM-1}}$)	(*aac(3)-II*), (*aac(3)-IIe*), *aac(6')-Ib*, (*aac(6')-IIc*), (*aadA1*), (*aadA2*), (*aph(3'')-Ib*), (*armA*)	(*dfrA1*), (*dfrA12*), (*dfrA19*), *sul1*, (*sul2*), (*qnrA1*), *fosA*, (*tet(D)*), (*mphE*), (*msrE*), (*mcr-9.1*), (*arr*)	12.8
**ST1762**	In238	2		*bla*$_{\text{VIM-4}}$, *bla*$_{\text{CTX-M-3}}$, *bla*$_{\text{TEM-1}}$	*aac(3)-IId*, *aac(6')-Ib*, *aadA1*, *aadA2*, *aph(3'')-Ib*, *aph(6)-Id*, *armA*	(*dfrA1*), (*dfrA12*), *dfrA19*, *sul1*, *sul2*, *qnrA1*, *fosA*, *tet(D)*, *catA2*, *mphE*, *msrE*, *mcr-9.1*	22.0
ST90	In238a+ **In2241**	2		*bla*$_{\text{VIM-4}}$, *bla*$_{\text{IMP-19}}$, *bla*$_{\text{LAP-2}}$, *bla*$_{\text{TEM-1}}$	*aac(3)-II*, *aac(6')-Ib*, *aadA1b*, *aadA2*, (*aph(3')-Ia*), *aph(3'')-Ib*, *aph(6)-Id*	*dfrA19*, *sul1*, *fosA*, *qnrS1*, *catB2*, *arr*	17.5
	In916	1		*bla*$_{\text{VIM-1}}$, *bla*$_{\text{CTX-M-15}}$, *bla*$_{\text{SHV-12}}$, *bla*$_{\text{OXA-1}}$, *bla*$_{\text{TEM-1}}$	*aac(3)-IIe*, *aac(6')-Ib*, *aadA1*, *aph(3')-XV*	*dfrA14*, *sul1*, *sul2*, *qnrB1*, *qnrS1*,*tet(A)*, *fosA*, *catB2*	17.0
	**In2240**	1		*bla*$_{\text{VIM-1}}$	*aac(6')-Ib*, *aadA1*, *aph(3'')-Ib*, *aph(6)-Id*, *aph(3')-XV*	*sul1*, *sul2*, *qnrS1*, *fosA*, *catB2*	11.0
	In237a	1		*bla*$_{\text{VIM-1}}$, *bla*$_{\text{OXA-1}}$, *bla*$_{\text{TEM-1}}$	*aac(3)-IId*, *aac(6')-Ib*, *aph(3'')-Ib*, *aph(6)-Id*	*dfrA14*, *sul1*, *sul2*, *qnrB1*, *fosA*, *tet(D)*, *mphE*, *msrE*	15.0
ST91	In1008	2		*bla*$_{\text{VIM-2}}$, *bla*$_{\text{OXA-1}}$, *bla*$_{\text{TEM-1}}$	*aac(3)-IId*, *aac(6')-Ib*, *aadA1*, *aph(3'')-Ib*, *aph(6)-Id*, *armA*	*dfrA14*, *sul1*, *sul2*, *qnrB1*, *fosA*, *tet(A)*	16.0

taxa/ST	integron	n isolates	remarks	acquired AMR genes[a]: resistance to β-lactams	acquired AMR genes[a]: resistance to aminoglycosides	acquired AMR genes[a]: resistance to other groups	n AMR genes/ isolate
	In70	3		*bla*$_{\text{VIM-1}}$, (*bla*$_{\text{SHV-12}}$), (*bla*$_{\text{LAP-2}}$)	*aac(6')-Ib*, (*aac(6')-Ib-cr*), *aadA1*, (*aadA2*), (*aadA16*), *ant(2'')-Ia*, (*aph(3')-Ia*), *aph(3')-VIa*, *aph(3'')-Ib*, *aph(6)-Id* , *aph(3')-XV*	(*dfrA14*), (*dfrA27*), *sul1*, *sul2*, (*qnrB6*), *qnrS1*, *fosA*, *tet(A)*, (*arr-3*), *catB2*, (*floR*)	21.0
	In916	1		*bla*$_{\text{VIM-1}}$, *bla*$_{\text{CTX-M-15}}$, *bla*$_{\text{OXA-1}}$, *bla*$_{\text{SHV-12}}$	*aac(3)-IIe* , *aac(6')-Ib*, *aadA1*, *ant(2'')-Ia*, *aph(3')-VIa*, *aph(3'')-Ib*, *aph(6)-Id* , *aph(3')-XV*	*dfrA14*, *sul1*, *sul2*, *qnrB1*, *qnrS1*, *fosA*, *tet(A)*, *catB2*	20.0
ST93	In238	2		*bla*$_{\text{VIM-4}}$, *bla*$_{\text{SHV-5}}$, (*bla*$_{\text{LAP-2}}$), (*bla*$_{\text{TEM-1}}$)	*aac(3)-Ia*, *aac(6')-Ib*, (*aadA1*)	(*dfrA1*), *sul1*, (*qnrS1*), *fosA*, (*tet(B)*), (*tet(G)*), (*catA1*), (*floR2*), *oqxB*	12.5
	In1008	1		*bla*$_{\text{VIM-2}}$, *bla*$_{\text{CTX-M-3}}$, *bla*$_{\text{TEM-1}}$	*aac(3)-IId*, *aac(6')-Ib*, *armA*	*sul1*, *fosA*, *oqxB*	9.0
ST106	In916	3		*bla*$_{\text{VIM-1}}$, *bla*$_{\text{CTX-M-15}}$, *bla*$_{\text{OXA-1}}$, *bla*$_{\text{TEM-1}}$	*aac(6')-Ib*, (*aac(6)-Id*), *aadA1*, *aph(3')-XV*	*dfrA14*, *sul1*, *sul2*, *qnrB1*, *fosA*, *tet(A)*, *catB2*, (*oqxB*)	16.0
ST110	In916	1		*bla*$_{\text{VIM-1}}$	*aac(6')-Ib*, *aadA1*, *aph(3'')-Ib*, *aph(6)-Id*, *aph(3')-XV*	*sul1*, *sul2*, *qnrS1*, *fosA*, *catB2*, *oqxB*	12.0
ST116	**In2240**	1		*bla*$_{\text{VIM-1}}$	*aac(6')-Ib*, *aadA1*, *aph(3'')-Ib*, *aph(6)-Id*, *aph(3')-XV*	*sul1*, *sul2*, *qnrS1*, *fosA*, *catB2*	11.0
ST134	In238	9	outbreak IX	*bla*$_{\text{VIM-4}}$, *bla*$_{\text{CTX-M-15}}$, *bla*$_{\text{OXA-1}}$	*aac(3)-IIe*, *aac(6')-Ib*, *aac(6')-Ib-cr*, *aadA1*	*dfrA14*, (*sul1*), *qnrB1*, (*catA1*), *fosA*, *tet(A)*, *oqxB*	14.0
	In238a	2		*bla*$_{\text{VIM-4}}$, *bla*$_{\text{CTX-M-15}}$, *bla*$_{\text{OXA-1}}$, *bla*$_{\text{TEM-1}}$	*aac(3)-IIe*, *aac(6')-Ib*, *aac(6')-Ib-cr*, *aadA1*, *aph(3'')-Ib*, *aph(6)-Id*	(*dfrA12*), (*dfrA14*), *sul1*, *sul2*, *qnrB1*, *fosA*, *tet(A)*, *oqxB*	18.0
	In916	2		*bla*$_{\text{VIM-1}}$, *bla*$_{\text{CTX-M-15}}$, *bla*$_{\text{SHV-12}}$, *bla*$_{\text{OXA-1}}$, *bla*$_{\text{TEM-1}}$	*aac(3)-IIe*, *aac(6')-Ib*, *aadA1*, *aph(3')-XV*	*dfrA14*, *sul1*, *sul2*, *qnrB1*, *qnrS1*, *fosA*, *tet(A)*, *catB2*	18.0
ST175	In238a	2		*bla*$_{\text{VIM-4}}$, *bla*$_{\text{CTX-M-15}}$, *bla*$_{\text{OXA-1}}$, *bla*$_{\text{TEM-1}}$	*aac(3)-IIe*, *aac(6')-Ib*, *aac(6')-Ib-cr*, *aadA1*, *aph(3'')-Ib*, *aph(6)-Id*	*dfrA14*, *sul1*, *sul2*, *qnrB1*, *fosA*, *tet(A)*	17.0
	In916	1		*bla*$_{\text{VIM-1}}$, *bla*$_{\text{CTX-M-15}}$, *bla*$_{\text{SHV-12}}$, *bla*$_{\text{OXA-1}}$, *bla*$_{\text{TEM-1}}$	*aac(3)-IIe*, *aac(6')-Ib*, *aadA1*, *aph(3'')-Ib*, *aph(3')-XV*	*dfrA14*, *sul1*, *sul2*, *qnrB1*, *qnrS1*, *fosA*, *tet(A)*, *catB2*	18.0
ST184	In916	1		*bla*$_{\text{VIM-1}}$, *bla*$_{\text{GES-7}}$, *bla*$_{\text{PER-2}}$, *bla*$_{\text{TEM-1}}$	*aac(6')-Ib*, *aadA1*, *aph(3')-XV*	*dfrA8*, *dfrB3*, *sul1*, *qnrS1*, *fosA*, *tet(C)*, *catA1*, *catB2*, *ereA*	16.0
ST517	In1444	1		*bla*$_{\text{VIM-20}}$, *bla*$_{\text{CTX-M-3}}$, *bla*$_{\text{OXA-1}}$, *bla*$_{\text{TEM-1}}$	*aac(6')-Ib*, *armA*	*dfrA14*, *sul1*, *sul2*, *qnrB1*, *qnrE1*, *fosA*, *tet(A)*, *mphE*, *msrE*	15.0
ST533	In916	1		*bla*$_{\text{VIM-1}}$, *bla*$_{\text{SHV-12}}$	*aac(6')-Ib*, *aadA1*, *aph(3'')-Ib*, *aph(6)-Id*, *aph(3')-XV*	*dfrA14*, *sul1*, *sul2*, *qnrS1*, *fosA*, *catB2*, *oqxB*	14.0
ST953	In238a	1		*bla*$_{\text{VIM-4}}$, ***bla***$_{\textbf{CTX-M-258}}$,[b] *bla*$_{\text{OXA-1}}$	*aac(3)-IId*, *aac(6')-Ib*, *aac(6')-Ib-cr*, *aadA1*, *aph(3'')-Ib*, *aph(6)-Id*	*dfrA14*, *sul1*, *sul2*, *qnrB1*, *fosA*, *tet(A)*	15.0
**ST1755**	In916	1		*bla*$_{\text{VIM-1}}$, *bla*$_{\text{SHV-12}}$	*aac(6')-Ib*, *aadA1*, *aph(3'')-Ib*, *aph(6)-Id*, *aph(3')-XV*	*sul1*, *sul2*, *qnrS1*, *fosA*, *catB2*	12.0
**ST1758**	In916	6		*bla*$_{\text{VIM-1}}$, *bla*$_{\text{CTX-M-15}}$, *bla*$_{\text{SHV-12}}$, (*bla*$_{\text{OXA-1}}$), (*bla*$_{\text{TEM-1}}$)	(*aac(3)-IIe*), *aac(6')-Ib*, *aadA1*, (*aph(3'')-Ib*), (*aph(6)-Id*), *aph(3')-XV*	*dfrA14*, *sul1*, (*sul2*), (*qnrB1*), (*qnrS1*), (*fosA*), (*tet(A)*), *catB2*, (*oqxA*), (*oqxB*)	17.0
E. hormaechei subsp. *xiangfangensis*							
ST66	In916	8	outbreak VII	*bla*$_{\text{VIM-1}}$, (*bla*$_{\text{CTX-M-15}}$), (*bla*$_{\text{SHV-12}}$), *bla*$_{\text{OXA-1}}$, (*bla*$_{\text{TEM-1}}$)	(*aac(3)-IIe*), *aac(6')-Ib*, *aadA1*, (*aph(3'')-Ib*), (*aph(6)-Id*), *aph(3')-XV*	*dfrA14*, *sul1*, *sul2*, (*qnrB1*), (*qnrS1*), (*fosA*), (*catA2*), *catB2*, (*oqxB*)	16.9
ST66	In916	10	outbreak VIII	*bla*$_{\text{VIM-1}}$, (*bla*$_{\text{CTX-M-15}}$), (*bla*$_{\text{OXA-1}}$), (*bla*$_{\text{TEM-1}}$)	(*aac(3)-IIe*), *aac(6')-Ib*, *aadA1*, (*aac(6')-Ib-cr*), (*aph(3'')-Ib*), *aph(3')-XV*	(*dfrA14*), *sul1*, (*sul2*), (*qnrB1*), (*qnrS1*), *fosA*, (*tet(A)*), *catB2*, (*oqxA*), (*oqxB*)	16.3
**ST1754**	In916	1		*bla*$_{\text{VIM-1}}$, *bla*$_{\text{SHV-12}}$, ***bla***$_{\textbf{FOX-20}}$[b]	*aac(6')-Ib*, *aadA1*, *aph(3'')-Ib*, *aph(6)-Id*, *aph(3')-XV*	*dfrA14*, *sul1*, *sul2*, *qnrB19*, *qnrS1*, *fosA*, *catB2*, *oqxB*	16.0
ST92	In238	2		*bla*$_{\text{VIM-4}}$, *bla*$_{\text{CTX-M-15}}$, *bla*$_{\text{OXA-1}}$, *bla*$_{\text{TEM-1}}$	*aac(3)-IIe*, *aac(6')-Ib*, *aph(3'')-Ib*, *aph(6)-Id*	*dfrA14*, *sul1*, *sul2*, *qnrB1*, *fosA*, *tet(A)*, *tet(D)*, (*oqxB*), (*mcr-9.1*)	17.0

taxa/ST	integron	n isolates	remarks	acquired AMR genes[a]			n AMR genes/ isolate
				resistance to β-lactams	resistance to aminoglycosides	resistance to other groups	
ST114	In238	2		bla_{VIM-4}, ($bla_{CTX-M-3}$), ($bla_{CTX-M-15}$), (bla_{OXA-1}), (bla_{TEM-1})	(*aac(3)-IIe*), *aac(6')-Ib*, (*aadA1*), (*aadA2*), (*aph(3'')-Ib*), (*aph(6)-Id*), (*armA*)	(*dfrA12*), (*dfrA14*), *sul1*, (*sul2*), (*qnrB1*), *fosA*, (*tet(A)*), (*mphE*), (*msrE*), (*oqxB*)	14.0
	In916	1		bla_{VIM-1}, $bla_{CTX-M-15}$, bla_{OXA-1}, bla_{TEM-1}	*aac(3)-IIe, aac(6')-Ib, aadA1, aph(3')-XV*	*dfrA14, sul1, sul2, qnrB1, qnrS1, fosA, tet(A), catB2*	16.0
ST121	In916	24	outbreak V	bla_{VIM-1}, $bla_{CTX-M-15}$, (bla_{SHV-12}), (bla_{OXA-1}), (bla_{TEM-1})	(*aac(3)-Ia*), (*aac(3)-IIe*), *aac(6')-Ib*, *aadA1*, *aph(3')-Ia*, (*aph(3'')-Ib*), (*aph(6)-Id*), *aph(3')-XV*	(*sat2*), (*dfrA1*), (*dfrA14*), (*sul1*), (*sul2*), (*qnrB1*), (*qnrS1*), (*fosA*), (*tet(A)*), (*catA1*), *catB2*, (*oqxA*), (*oqxB*), (*mphA*)	18.9
**ST1756**	In916	3		bla_{VIM-1}, $bla_{CTX-M-15}$, bla_{SHV-12}, (bla_{OXA-1}), (bla_{TEM-1})	*aac(6')-Ib, aadA1, aph(3')-Ia, aph(3'')-Ib, aph(6)-Id, aph(3')-XV*	*sat2, dfrA1*, (*dfrA14*), (*sul1*), *sul2*, (*qnrB1*), *fosA, tet(A), catB2*, (*oqxA*), (*oqxB*)	20.0
ST121	In238a	6	outbreak VI	bla_{VIM-4}, $bla_{CTX-M-15}$, bla_{OXA-1}, bla_{TEM-1}	*aac(3)-IId, aac(6')-Ib*, (*aac(6')-Ib-cr*), (*aadA1*), *aadA2, aph(3')-Ia, aph(3'')-Ib, aph(6)-Id*	*sat2, dfrA1*, (*dfrA12*), *dfrA14, sul1, sul2, qnrB1, fosA, tet(A)*, (*catA1*), (*oqxB*), (*mphA*)	20.5
	In238	3		bla_{VIM-4}, $bla_{CTX-M-15}$, bla_{OXA-1}, bla_{TEM-1}	(*aac(3)-IIe*), *aac(6')-Ib*, (*aac(6')-Ib-cr*), *aph(3')-Ia, aph(3'')-Ib, aph(6)-Id*, (*armA*)	*sat2, dfrA1, dfrA14, sul1*, (*sul2*), *qnrB1, fosA*, (*tet(A)*), (*mphE*), (*msrE*), (*oqxA*), *oqxB*	20.3
	In2016-like	1		bla_{VIM-4}, $bla_{CTX-M-15}$, bla_{OXA-1}, bla_{OXA-10}, bla_{TEM-1}	*aac(3)-IId, aph(3')-Ia, aph(3')-VIb*	*sat2, arr-3, dfrA1, dfrA14, sul1, sul2, fosA, tet(A), oqxA*	17.0
	**In2242**	2		bla_{VIM-2}, ($bla_{CTX-M-15}$), (bla_{OXA-1}), (bla_{TEM-1})	*aac(3)-I*, (*aac(6')-Ib-cr*), (*aadA2*), *aadA6-10*, (*aph(3')-Ia*), (*aph(3'')-Ib*), *aph(6)-Id*, (*armA*)	(*sat2*), (*dfrA1*), *dfrA14, sul1*, (*sul2*), *fosA, tet(A)*, (*mphE*), (*msrE*), (*oqxA*), (*oqxB*)	16.0
ST407	In916	2		bla_{VIM-1}, $bla_{CTX-M-15}$, bla_{SHV-12}, bla_{OXA-1}, bla_{TEM-1}	*aac(6')-Ib, aadA1, aph(3')-XV*	*dfrA14, sul1, sul2, qnrB1, qnrS1, fosA, tet(A), catB2, oqxB*	18.0
	In238	2		bla_{VIM-4}, $bla_{CTX-M-15}$, bla_{OXA-1}, bla_{TEM-1}	(*aac(3)-I*), *aac(3)-IIe, aac(6')-Ib, aac(6')-Ib-cr*, (*aadA1*), (*aadA2*), (*aadA6*), (*aph(3')-Ia*), *aph(3'')-Ib, aph(6)-Id*, (*armA*)	(*sat2*), (*dfrA1*), (*dfrA12*), *dfrA14, sul1, sul2, qnrB1, fosA, tet(A)*, (*mphE*), (*msrE*), (*oqxB*)	22.0
ST527	In611-like	1		bla_{VIM-1}, bla_{GES-7}, bla_{SHV-12}, bla_{LAP-2}	*aac(3)-Ia, aac(6')-Ib, aadA1,aph(6)-Id, aph(3')-XV*	*dfrA14, dfrB3, sul1, qnrS1, fosA, tet(A), catB2, oqxB*	17.0
**ST1757**	In916	3		bla_{VIM-1}, $bla_{CTX-M-15}$, (bla_{SHV-12}), bla_{OXA-1}	*aac(3)-IIe, aac(6')-Ib, aadA1, aph(3'')-Ib, aph(6)-Id, aph(3')-XV*	(*sat2*), (*dfrA1*), *dfrA14, sul1, sul2*, (*qnrB1*), *qnrS1, fosA, tet(A), catB2*, (*oqxA*)	19.7
E. hormaechei subsp. *hoffmannii*							
ST78	**In2241**	2		bla_{IMP-19}, bla_{TEM-1}	(*aac(3)-II*), *aac(6')-Ib, aadA1b*, (*aadA2*), *aph(3')-Ia, aph(3'')-Ib, aph(6)-Id*	*dfrA15*, (*dfrA19*), (*dfrA25*), *sul1, fosA, catB2, oqxB*, (*arr*)	15.5
	In110	1		bla_{VIM-1}, **$bla_{CTX-M-257}$**,[b] bla_{LAP-2}, bla_{TEM-1}	*aac(3)-IId, aac(6')-Ib, aadA1, armA*	*dfrA15, sul1, qnrS1, fosA, mphE, msrE, oqxB*	15.0
	In238	1		bla_{VIM-4}	*aac(6')-Ib, aac(6')-Ib-cr*	*dfrA25, sul1, qnrB2, fosA, oqxB*	8.0
ST97	In916	1		bla_{VIM-1}, bla_{SHV-12}	*aac(6')-Ib, aadA1, aadA16, aph(3'')-Ib, aph(6)-Id, aph(3')-XV*	*dfrA14, dfrA27, sul1, sul2, qnrB6, qnrS1, fosA, catB2, oqxB, arr-3*	18.0
ST102	In916	1		bla_{VIM-1}, $bla_{CTX-M-9}$, bla_{SHV-12}	*aac(6')-Ib, aadA1, aadA2, aph(3'')-Ib, aph(6)-Id, aph(3')-XV*	*dfrA16, sul1, sul2, qnrA1, fosA, tet(A), catB2, oqxB*	17.0
	In238	1		bla_{VIM-4}, $bla_{CTX-M-15}$, bla_{OXA-1}, bla_{TEM-1}	*aac(3)-IIe, aac(6')-Ib, aac(6')-Ib-cr, aadA1, aph(3'')-Ib, aph(6)-Id*	*dfrA14, sul1, sul2, qnrB1, fosA, tet(A), oqxB*	17.0
ST104	In916	7		bla_{VIM-1}, bla_{SHV-12}, (bla_{OXA-1}), (bla_{TEM-1})	*aac(6')-Ib*, (*aac(6')-Ib-cr*), *aadA1*, (*aph(3'')-Ib*), (*aph(6)-Id*), *aph(3')-XV*	*dfrA14, sul1*, (*sul2*), (*qnrA1*), (*qnrS1*), *fosA*, (*tet(D)*), (*catA2*), *catB2*, (*catB3*), *oqxB*, (*arr-3*)	17.6
ST118	In916	1		bla_{VIM-1}, $bla_{CTX-M-15}$, bla_{SHV-12}, bla_{OXA-1}, bla_{TEM-1}	*aac(3)-IIe, aac(6')-Ib, aadA1, aph(3')-XV*	*dfrA14, sul1, sul2, qnrB1, qnrS1, fosA, tet(A), catB2, oqxB*	18.0

taxa/ST	integron	n isolates	remarks	acquired AMR genes[a]			n AMR genes/ isolate
				resistance to β-lactams	resistance to aminoglycosides	resistance to other groups	
ST135	In238	1		$bla_{\text{VIM-4}}$	*aac(6')-Ib, aph(3')-Ia*	*sul1, fosA, oqxB*	6.0
ST145	In238	1		$bla_{\text{VIM-4}}$, $bla_{\text{SHV-12}}$, $bla_{\text{OXA-10}}$	*aac(6')-Ib, aadA1, aadA2, ant(2'')-Ia*	*dfrA19, sul1, sul2, qnrA1, fosA, catA2, catB3, cmlA1, oqxB, mcr-9.1*	17.0
ST173	In916	2		$bla_{\text{VIM-1}}$, ($bla_{\text{CTX-M-3}}$), ($bla_{\text{CTX-M-14}}$), $bla_{\text{SHV-12}}$, ($bla_{\text{TEM-1}}$)	(*aac(3)-II*), (*aac(6')-IIc*), *aac(6')-Ib, aadA1*, (*aph(3'')-Ib*), (*aph(6)-Id*), *aph(3')-XV*	(*dfrA14*), (*dfrA19*), *sul1, sul2*, (*qnrA1*), *qnrS1, fosA, catB2, oqxB*, (*mcr-9.1*), (*arr*)	18.0
	In238	1		$bla_{\text{VIM-4}}$, $bla_{\text{SHV-5}}$, $bla_{\text{LAP-2}}$	*aac(6')-Im, aac(6')-Ib, aph(2'')-IIa,*	*sul1, qnrS1, fosA, tet(B), catA1, oqxB*	12.0
	In238a	1		$bla_{\text{VIM-4}}$, $bla_{\text{SHV-12}}$, $bla_{\text{OXA-10}}$	*aac(6')-Ib, aadA1, aadA2, ant(2'')-Ia*	*dfrA19, sul1, sul2, qnrA1, fosA, catA2, catB3, cmlA1, oqxB, mcr-9.1*	17.0
ST316	In238	3		$bla_{\text{VIM-4}}$, $bla_{\text{CTX-M-15}}$, $bla_{\text{OXA-1}}$, $bla_{\text{TEM-1}}$	*aac(3)-IIe, aac(6')-Ib, aac(6')-Ib-cr, aadA1, aph(3'')-Ib, aph(6)-Id*	*dfrA14, sul1, sul2, qnrB1, fosA, tet(A), oqxB*	18.0
	In916	1		$bla_{\text{VIM-1}}$, $bla_{\text{CTX-M-15}}$	*aac(6')-Ib, aadA1, aph(3')-XV*	*sul1, fosA, catB2, oqxB*	9.0
ST381	In916	1		$bla_{\text{VIM-1}}$, $bla_{\text{SHV-12}}$	*aac(6')-Ib, aadA1, aph(3')-XV*	*dfrA14, sul1, fosA, catB2, oqxB*	10.0
ST485	In238	2		$bla_{\text{VIM-4}}$, ($bla_{\text{SHV-12}}$), ($bla_{\text{OXA-10}}$)	*aac(6')-Ib*, (*aadA1*), (*aadA2*), (*ant(2'')-Ia*), (*aph(3'')-Ib*), (*aph(6)-Id*), (*armA*)	(*dfrA19*), *sul1, sul2*, (*qnrA1*), *fosA, catB3*, (*cmlA1*), (*mphE*), (*msrE*), *oqxB, mcr-9.1*	15.5
ST764	In916	1		$bla_{\text{VIM-1}}$, $bla_{\text{CTX-M-15}}$, $bla_{\text{OXA-1}}$, $bla_{\text{TEM-1}}$	*aac(3)-IIe, aac(6')-Ib, aadA1, aph(3')-XV*	*dfrA14, sul1, sul2, qnrB1, qnrS1, fosA, tet(A), catB2, oqxB*	17.0
ST1641	In916	1		$bla_{\text{VIM-1}}$, $bla_{\text{SHV-12}}$	*aac(6')-Ib, aadA1, aph(3'')-Ib, aph(6)-Id, aph(3')-XV*	*dfrA14, sul1, sul2, qnrS1, fosA, catB2, oqxB*	14.0
**ST1753**	In916	3		$bla_{\text{VIM-1}}$, $bla_{\text{SHV-12}}$	*aac(6')-Ib, aadA1, aph(3'')-Ib, aph(6)-Id, aph(3')-XV*	*dfrA14, sul1, sul2, qnrS1, fosA, catB2, oqxB*	15.0
**ST1759**	In916	2		$bla_{\text{VIM-1}}$, $bla_{\text{CTX-M-15}}$, $bla_{\text{OXA-1}}$, $bla_{\text{TEM-1}}$	*aac(3)-IIe, aac(6')-Ib, aadA1, aph(3')-XV*	*dfrA14, sul1, sul2*, (*qnrB1*), *qnrS1, fosA, tet(A), catB2, oqxB*	17.5
E. hormaechei subsp. *oharae*							
ST68	In238	1		$bla_{\text{VIM-4}}$, $bla_{\text{CTX-M-3}}$, $bla_{\text{TEM-1}}$	*aac(3)-IId, aac(6')-Ib, armA*	*sul1, mphE, msrE, oqxB*	10.0
ST94	In70	1		$bla_{\text{VIM-1}}$, $bla_{\text{SHV-12}}$, $bla_{\text{OXA-10}}$	*aac(6')-Ib, aadA1, aadA2, ant(2'')-Ia, aph(3')-XV*	*sat2, dfrA1, dfrA14, dfrA19, sul1, sul2, qnrS1, catA2, catB2, catB3, cmlA1, mcr-9.1*	20.0
	In238	1		$bla_{\text{VIM-4}}$	*aac(6')-Ib, aadA1, aph(3'')-Ib, aph(6)-Id, armA*	*sat2, dfrA1, sul1, qnrS1, tet(A), mphE, msrE, oqxA*	14.0
ST108	In1008	8		$bla_{\text{VIM-2}}$, ($bla_{\text{CTX-M-9}}$)	*aac(6')-Ib*, (*aadA2*)	(*dfrA16*), *sul1, tet(A), oqxB*	6.7
E. hormaechei subsp. *hormaechei*							
ST528	In238	1		$bla_{\text{VIM-4}}$, $bla_{\text{CTX-M-15}}$, $bla_{\text{OXA-1}}$	*aac(3)-IIe, aac(6')-Ib*	*dfrA14, qnrB1, tet(A)*	8.0
E. roggenkampii							
ST95	In238	1		$bla_{\text{VIM-4}}$	*aac(6')-Ib*	*sul1, fosA, tet(A), oqxB*	6.0
ST96	In238	4		$bla_{\text{VIM-4}}$, ($bla_{\text{CTX-M-3}}$), ($bla_{\text{GES-7}}$), ($bla_{\text{SHV-12}}$), ($bla_{\text{OXA-1}}$), ($bla_{\text{OXA-10}}$), ($bla_{\text{TEM-1}}$)	(*aac(3)-IId*), *aac(6')-Ib*, (*aadA2*), (*ant(2'')-Ia*), (*aph(3')-Ia*), (*aph(3')-Ib*), (*aph(6)-Id*), (*armA*)	(*dfrA12*), (*dfrA19*), (*dfrB3*), *sul1*, (*qnrS2*), *fosA*, (*catA2*), (*catB3*), (*mphE*), (*msrE*), (*oqxA*), (*oqxB*), (*mcr-9.1*), (*arr-3*)	14.5
ST166	In916	1		$bla_{\text{VIM-1}}$	*aac(6')-Ib, aadA1, aph(3'')-Ib, aph(6)-Id, aph(3')-XV*	*sul1, sul2, qnrS1, fosA, catB2, oqxB*	12.0
ST523	In238	1		$bla_{\text{VIM-4}}$	*aac(6')-Ib, armA*	*sul1, fosA, mphE, msrE*	7.0
**ST1761**	In238	1		$bla_{\text{VIM-4}}$	*aac(6')-Ib*	*sul1, fosA, oqxB*	5.0
E. asburiae							
ST23	In238	1		$bla_{\text{VIM-4}}$	*aac(6')-Ib*	*sul1, fosA*	4.0
ST25	In238a	1		$bla_{\text{VIM-4}}$, $bla_{\text{CTX-M-3}}$, $bla_{\text{OXA-1}}$, $bla_{\text{TEM-1}}$	*aac(6')-Ib, aadA1, aadA2, aph(3')-VIa*	*dfrA14, qnrB1, qnrS1, fosA, tet(A), tet(C), oqxB*	15.0
E. kobei							

taxa/ST	integron	n isolates	remarks	acquired AMR genes[a]: resistance to β-lactams	acquired AMR genes[a]: resistance to aminoglycosides	acquired AMR genes[a]: resistance to other groups	n AMR genes/ isolate
ST99	In238	1		*bla*$_{VIM-4}$, *bla*$_{SHV-5}$	*aac(6')-Im, aac(6')-Ib, aadA1, aph(2")-IIa, aph(3')-VI*	*dfrA1, sul1, fosA, tet(B), catA1, oqxB*	13.0
E. ludwigii							
ST1306	In916	1		*bla*$_{VIM-1}$, *bla*$_{SHV-12}$	*aac(6')-Ib, aadA1, aph(3")-Ib, aph(6)-Id, aph(3')-XV*	*dfrA14, sul1, sul2, qnrS1, fosA, catB2, oqxB*	14.0
E. mori							
**ST1760**	In238a	1		*bla*$_{VIM-4}$, *bla*$_{CTX-M-9}$	*aac(6')-Ib, aac(6')-Ib-cr, aadA2, ant(2")-Ia*	*dfrA16, sul1, qnrA1, qnrE1, fosA, oqxB*	12.0

[a] – symbols in parentheses refer to the genes that occurred not in all isolates of the corresponding genotypes.

[b] – new *bla*$_{CTX-M}$ and *bla*$_{FOX}$ genes are indicated in bold; nucleotide sequences of new genes are available under the following GenBank accession numbers: *bla*$_{CTX-M-256}$, OP081688; *bla*$_{CTX-M-258}$, OP346113; *bla*$_{FOX-20}$, OP297845; *bla*$_{CTX-M-257}$, OP297846.

Appendix Table 10. Resistomes of the isolates subjected to the long-read WGS analysis

Isolate (ST)	Plasmids with *bla*$_{VIM}$ gene	Other plasmids	AMR genes located on short contigs not assigned to individual plasmids or chromosome	Chromosome
743/14 (ST121)	**IncA:** *bla*$_{VIM-1}$, *bla*$_{SHV-12}$, *aac(6')-Ib, aadA1, aph(3")-Ib, aph(3')-XV, aph(6)-Id, mphA, catB2, sul1, sul2, qnrS1, dfrA14*	**IncHI2+HI2A:** *bla*$_{OXA-1}$, *aac(6')-Ib-cr, aadA1, catA1, qnrB1, tet(A), dfrA14*	-	*bla*$_{CTX-M-15}$, *bla*$_{TEM-1}$ (x2), *aph(3')-Ia, aph(6)-Id, aph(3'')-Ib, sul2, dfrA1, fosA, oqxB, sat2*
5955/16 (ST66)	**IncA:** *bla*$_{VIM-1}$, *aac(6')-Ib*, *aadA1*, *aph(3")-Ib, aph(3')-XV, aph(6)-Id*, *catB2*, *sul1*, *sul2, qnrS1,*	**IncFII+FIB:** *bla*$_{OXA-1}$, *aac(6')-Ib-cr, catA2, qnrB1, dfrA14*	-	*fosA, oqxB*
7753/18 (ST89)	**IncA:** *bla*$_{VIM-1}$, *bla*$_{SHV-12}$, *aac(6')-Ib*, *aph(3")-Ib, aadA1*, *aph(3')-XV, aph(6)-Id*, *mphA*, *catB2, sul1, sul2, dfrA14*	**IncFII+FIB:** *bla*$_{OXA-1}$, *aac(6')-Ib-cr, aadA1*, catB3, *catA1*, *tet(A)*	*bla*$_{CTX-M-15}$, *bla*$_{TEM-1}$, *aac(3)-IIe, aph(6)-Id*, *aph(3")-Ib, sul2*	*aadA1*, *dfrA1, fosA*
4969/09 (ST90)	**IncHI2+HI2A:** *bla*$_{VIM-4}$, *aac(6')-Ib*, *catA2, sul1, mcr-9.1*	-	*bla*$_{OXA-10}$*, aac(6')-Ib, aadA1, aadA2, ant(2")-Ia, catB3, sul1* (x2), *dfrA19, cmlA1*	*bla*$_{CTX-M-3}$ (x3), *fosA*
6234/09 (ST90)	**IncFII+FIA:** *bla*$_{VIM-4}$, *aac(6')-Ib*	-	-	*bla*$_{CTX-M-3}$ (2x), *bla*$_{TEM-1}$, *aac(3)-IIa*, *aph(3'')-Ib*, *aadA1*, *dfrA1*, *sul1* (2x), *fosA*
5435/13 (ST134)	**IncN3:** *bla*$_{VIM-4}$, *aac(6')-Ib, sul1*	**IncHI2+HI2A:** *bla*$_{CTX-M-15}$, *bla*$_{OXA-1}$, *aac(6')-Ib-cr, aac(3)-IIe, aadA1, catA1, qnrB1, tet(A), dfrA14*	-	*fosA, oqxB*
5713/17 (ST121)	**IncFIB:***bla*$_{VIM-4}$, *bla*$_{TEM-1}$, *aac(3)-IId*, *aac(6')-Ib, aadA2, mphA, sul1* (x2), *dfrA12*	**IncHI2+HI2A:** *bla*$_{OXA-1}$, *aac(6')-Ib-cr, aadA1, catA1, qnrB1, tet(A), dfrA14*	-	*bla*$_{CTX-M-15}$, *bla*$_{TEM-1}$ (x2), *aph(3')-Ia, aph(6)-Id, aph(3'')-Ib, sul2, dfrA1, fosA, oqxB, sat2*
8770/11 (ST89)	-	-	-	*bla*$_{CTX-M-3,}$ *bla*$_{VIM-40,}$ *aac(6')-Ib, aph(3'')-Ib, aph(3')-VI, aph(6)-Id, sul1, fosA*
2944/06 (ST89)	-	-	-	*bla*$_{CTX-M-3}$ (x2),*bla*$_{VIM-20}$, *aac(3)-IId, aac(6')-Ib, aph(3')-Ia, aadA2, armA* (x2), *sul1*(x2), *dfrA12, fosA, msr(E)* (x2), *mph(A), mph(E)* (x2)

Appendix Table 11. Replicon types of the *Enterobacter* spp. isolates

taxa/ST	integron	n isolates	replicon types[a]	n replicon types/ isolate
E. hormaechei subsp. *steigerwaltii*				
ST45	In238	7	(A), FIB, FII, HI2, HI2A, ColRNAI	5.3
	In916	2	A, FIB, (M1), Col440I, Col440II, ColRNAI	5.0
ST62	In916	1	A, N, Col440II, ColRNAI	4.0
ST89	In916	48	A, (FIA), (FII), (HI1A), (HI1B), (HI2), (HI2A), (M1), (Col440II), ColRNAI	4.5
	In238	3	(A), (HI2), (HI2A), (M1), ColRNAI	1.7
	In1654	1	FII, HI2, HI2A, M2, Col440II, ColRNAI	6.0
	**In2238**	1	HI2, HI2A	2.0
	In1445	7	(FII)	0.1
	In1008	2	M1, ColRNAI	2.0
	In1444	12	(FII), ColpVC	1.1
ST90	In238	107	(A), (FIA), (FIB), (FII), (HI1A), (HI1B), (HI2), (HI2A), (M1), (M2), (N), (U), (Col440II), (ColRNAI), (ColpVC), (ColpWES), (p0111)	4.1
	In238a	5	FIA, FII, (HI2), (HI2A), (Col440I), (Col440II), (ColRNAI)	3.6
	In238a; **In2241**	2	HI2, HI2A, N3, (Col440II), ColRNAI	4.5
	In916	1	A, HI2, HI2A, Col440II, ColRNAI	5.0
	**In2240**	1	A, FIB, Col440I, Col440II	4.0
	In237a	1	FIB, M2, ColRNAI	3.0
ST91	In1008	2	HI2, HI2A, M2	3.0
	In70	3	A, (N), (HI2), (Col440II), (ColRNAI), (ColpVC)	3.7
	In916	1	A	1.0
ST93	In238	2	FIB, (FII), (HI2), (HI2A), (R), Col440II, ColRNAI	5.0
	In1008	1	FII, M2, R, Col440II, ColRNAI	5.0
ST106	In916	1	A, (FIB), HI2, HI2A, Col440II, ColRNAI	5.3
ST110	In916	1	A, ColRNAI	2.0
ST116	**In2240**	1	A	1.0
ST134	In238	9	HI2, HI2A, N3, (ColRNAI)	3.1
	In238a	2	N3	1.0
	In916	2	A	1.0
ST175	In238a	2	HI2, HI2A, N3	3.0
	In916	1	A, HI2, HI2A	3.0
ST184	In916	1	A, HI2, HI2A, Q2	4.0
ST517	In1444	1	FIB, FII, M2, ColRNAI	4.0
ST533	In916	1	A	1.0
ST953	In238a	1	FIB, HI2, HI2A, ColpVC	4.0
**ST1755**	In916	1	A, FIB, FII, Col440I, Col440II	5.0
**ST1758**	In916	6	(A), (HI2), (HI2A), Col440I, Col440II, (ColRNAI)	5.2
**ST1762**	In238	2	FIA, FIB, (FII), HI2, HI2A, Col440II, ColRNAI	6.5
E. hormaechei subsp. *xiangfangensis*				
ST66	In916	18	A, (FIB), (FII), (HI2), (HI2A), (M1), (Col440II), ColRNAI	4.2
ST92	In238	2	FIB, FII, M1, Col440I, Col440II, ColRNAI	6.0
ST114	In238	2	(HI1A), (HI1B), (HI2), (HI2A), (M2), (N3), (Col440II), ColRNAI	4.5
	In916	1	A, FIB, HI2, HI2A, Col440II, ColRNAI	6.0
ST121	In916	24	A, FIB, (FII), (HI2), (HI2A), (M1), (R), (Col440II), (ColRNAI), (ColpVC)	5.0
	In238a	6	FIB, HI2, HI2A, ColRNAI	4.0
	In238	3	(C), FIB, HI2, HI2A, (N3), (ColRNAI), (ColpVC)	4.7

taxa/ST	integron	n isolates	replicon types[a]	n replicon types/ isolate
	In2016-like	1	C, FIB, FII, HI2, HI2A, Col440II, ColRNAI, ColRGK	8.0
	**In2242**	2	FIB, FII, HI2, HI2A, (M1), (Col440II), ColRNAI	6.0
ST407	In916	2	A, HI2, HI2A, ColRNAI	4.0
	In238	2	(C), HI2, HI2A, (N3), Col440II, ColRNAI	5.0
ST527	In611-like	1	A, M1, Q2, ColRNAI	4.0
**ST1754**	In916	1	A, Q2, Col440I	3.0
**ST1756**	In916	3	A, FIB, HI2, HI2A, Col440II, ColRNAI	6.0
**ST1757**	In916	3	A, FII, HI2, HI2A, (M1), (R), (Col440II), ColRNAI	6.7
E. hormaechei subsp. *hoffmannii*				
ST78	**In2241**	2	FIB, (FII), HI2, HI2A, Col440I, Col440II	5.5
	In110	1	FIB, M2, Col440II	3.0
	In238	1	FII, N3, ColRNAI	3.0
ST97	In916	1	A, N, Col440II, ColRNAI	4.0
ST102	In916	1	A, HI2, HI2A, Col440I, ColRNAI	5.0
	In238	1	FII, HI2, HI2A, N3, R, ColRNAI	6.0
ST104	In916	7	A, (FIB), (FIB_K), (FII), (HI1A), (HI1B), (N), (N2), (Col440II), ColRNAI	5.3
ST118	In916	1	A, FIB, Col440II, ColRNAI	4.0
ST135	In238	1	Col440I, Col440II, ColRNAI	3.0
ST145	In238	1	FIB, FII, HI2, HI2A, N3, ColRNAI	6.0
ST173	In916	2	A, (HI2), (HI2A), (M2), (N), Col440II, ColRNAI	5.0
	In238	1	A, Col440I, Col440II, ColRNAI	4.0
	In238a	1	HI2, HI2A, N, N3	4.0
ST316	In238	3	(FIB), HI2, HI2A, N3, R, ColRNAI	5.3
	In916	1	A, R, ColRNAI	3.0
ST381	In916	1	A, FIB, FII, R, Col440I, ColRNAI	6.0
ST485	In238	2	(C), FIB, (FII), HI2, (HI2A), (N3), (R), (Col440II), ColRNAI	6.0
ST764	In916	1	A, FIB, HI2, HI2A, ColRNAI	5.0
ST1641	In916	1	A, FIB, ColRNAI	3.0
**ST1753**	In916	3	A, ColRNAI	2.0
**ST1759**	In916	2	A, HI2, HI2A, ColRNAI	4.0
E. hormaechei subsp. *oharae*				
ST68	In238	1	ColRNAI	1.0
ST94	In70	1	A, HI2, HI2A	3.0
	In238	1	C, N, ColRNAI	3.0
ST108	In1008	8	FIB, (FII), (HI2), (HI2A), M1, R, Col440II, ColRNAI	6.4
ST528	In238	1	HI1A, HI1B, M1, ColRNAI	4.0
ST95	In238	1	FIB, FII, M1, Col440II, ColRNAI, pENTAS02	6.0
ST96	In238	4	(HI2), (HI2A), (M2), (Q2), (R), (U), Col440I, (ColRGK)	3.2
ST166	In916	1	A, FIB_K, HI1A, HI1B, Col440II, ColRNAI, pENTAS02	7.0
ST523	In238	1	C, Col440II, ColMG828, pKPC-CAV1321	4.0
**ST1761**	In238	1	FIB, FII, Col440II, ColRNAI, pKPC-CAV1321	5.0
E. asburiae				
ST23	In238	1	FIB, Col440II, ColYe4449, pENTAS02	4.0
ST25	In238a	1	FIB, FII, HI2, HI2A, N, Col440II, ColRNAI, pENTAS02	8.0
E. kobei				
ST99	In238	1	A, FIB_K, FII, Col440II, ColRNAI, pKPC-CAV1321	6.0
E. ludwigii				

taxa/ST	integron	n isolates	replicon types[a]	n replicon types/ isolate
ST1306	In916	1	A	1.0
E. mori				
**ST1760**	In238a	1	FII, HI2, HI2A, N3, Col440I, Col440II, ColRNAI	7.0

[a] – symbols in parentheses refer to the replicon types that occurred not in all isolates of the corresponding genotypes.

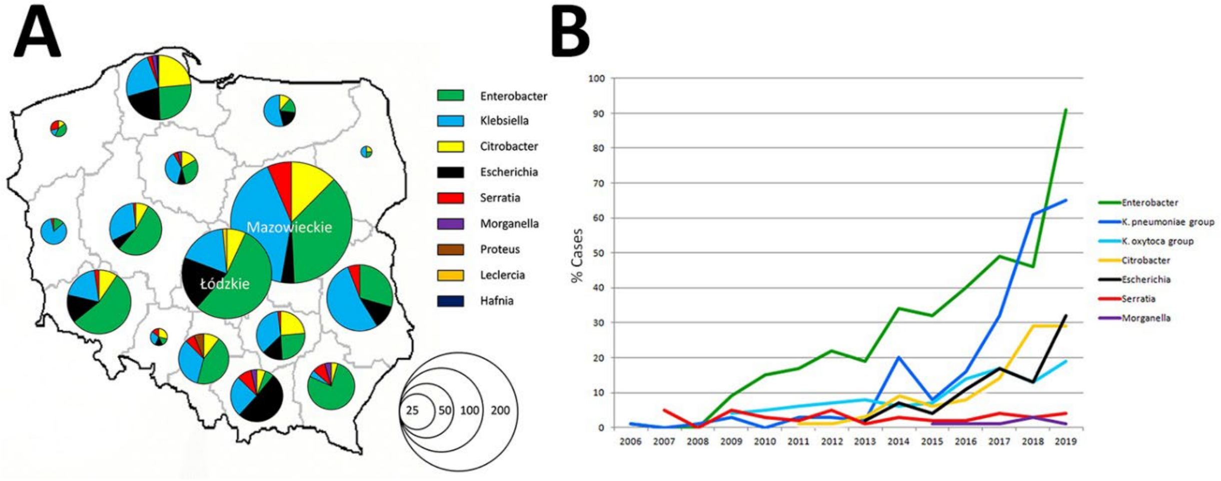

Appendix Figure 1. VIM/IMP-producing Enterobacterales in Poland; A) regional, and B) annual distribution

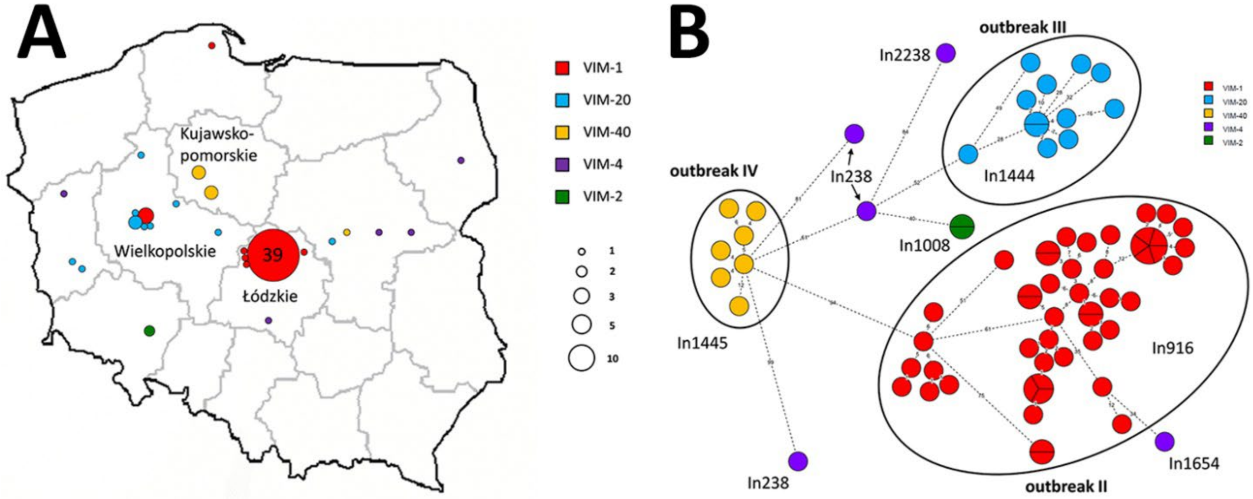

Appendix Figure 2. Geographic distribution and clonal analysis of *E. hormaechei* ST89 in Poland. A) Geographic distribution of the isolates shown on the map of the country with main administrative regions. Circles represent medical centres where the isolates were recorded. Sizes of the circles are proportional to numbers of cases. B) SNP-based minimum spanning tree of the isolates. Lengths of branches are related to numbers of SNPs between linked isolates. Numbers of SNPs are indicated above the branches or next to the dots.

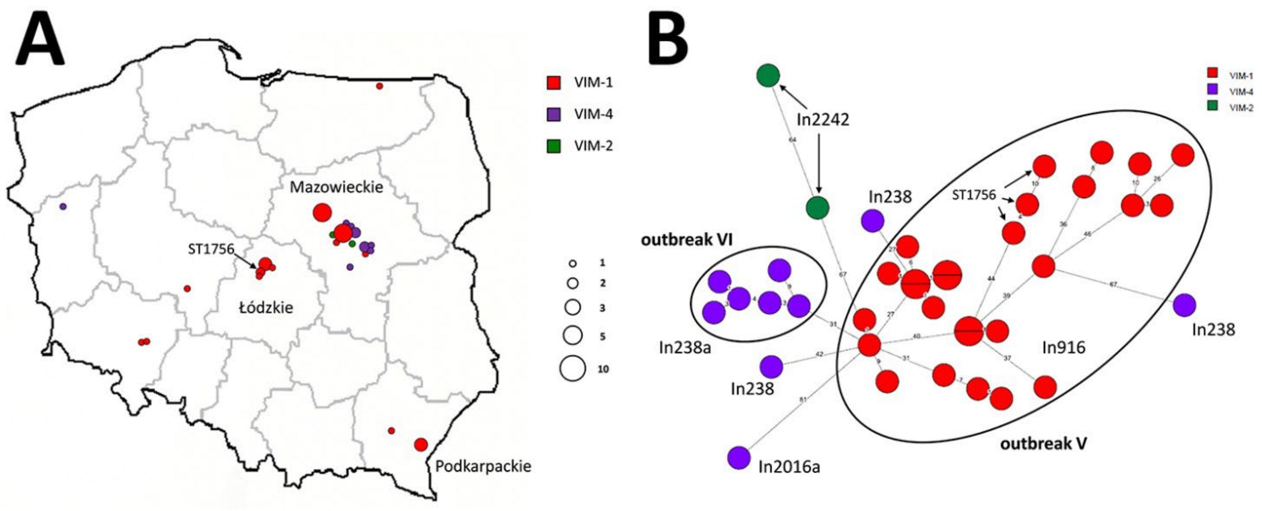

Appendix Figure 3. Geographic distribution and clonal analysis of *E. hormaechei* CC121 (ST121 and ST1756) in Poland. A) Geographic distribution of the isolates shown on the map of the country with main administrative regions. Circles represent medical centres where the isolates were recorded. Sizes of the circles are proportional to numbers of cases. B) SNP-based minimum spanning tree of the isolates. Lengths of branches are related to numbers of SNPs between linked isolates. Numbers of SNPs are indicated above the branches or next to the dots.

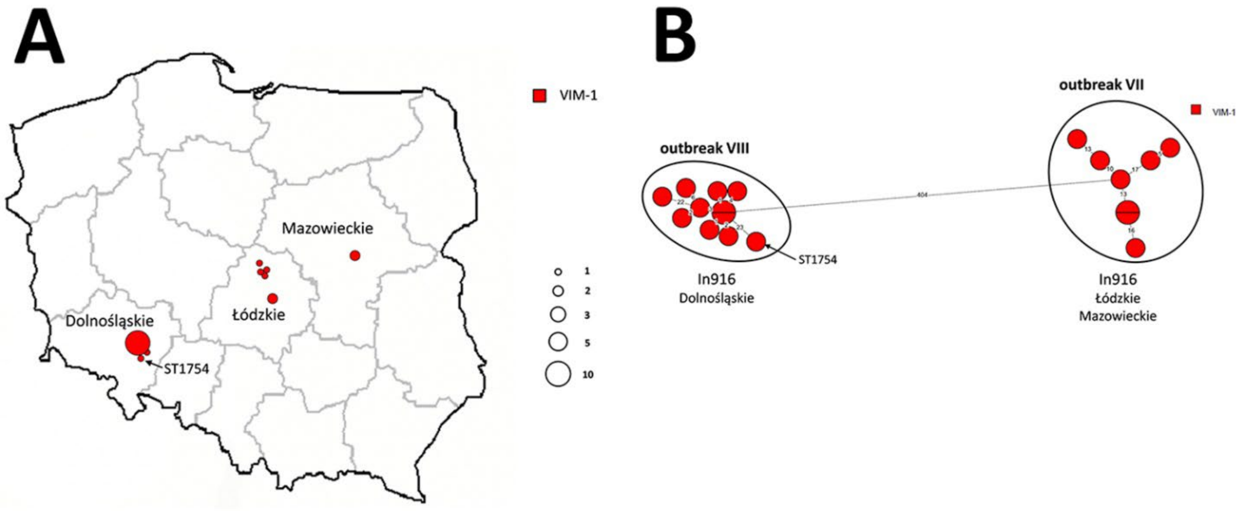

Appendix Figure 4. Geographic distribution and clonal analysis of *E. hormaechei* CG66 (ST66 and ST1754) in Poland. A) Geographic distribution of the isolates shown on the map of the country with main administrative regions. Circles represent medical centres where the isolates were recorded. Sizes of the circles are proportional to numbers of cases. B) SNP-based minimum spanning tree of the isolates. Lengths of branches are related to numbers of SNPs between linked isolates. Numbers of SNPs are indicated above the branches or next to the dots.

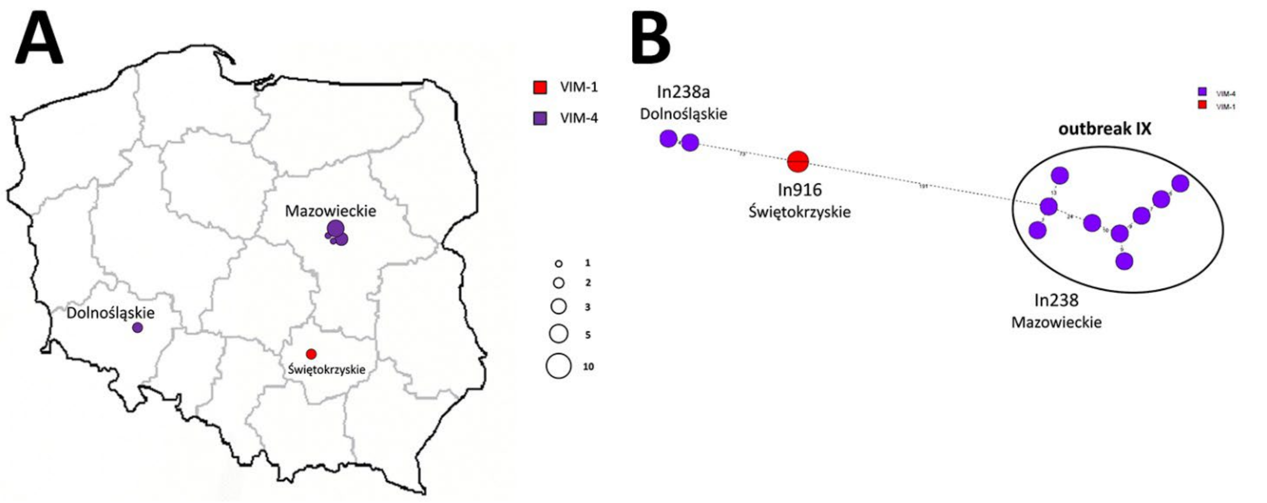

Appendix Figure 5. Geographic distribution and clonal analysis of *E. hormaechei* ST134 in Poland. A) Geographic distribution of the isolates shown on the map of the country with main administrative regions. Circles represent medical centres where the isolates were recorded. Sizes of the circles are proportional to numbers of cases. B) SNP-based minimum spanning tree of the isolates. Lengths of branches are related to numbers of SNPs between linked isolates. Numbers of SNPs are indicated above the branches or next to the dots.

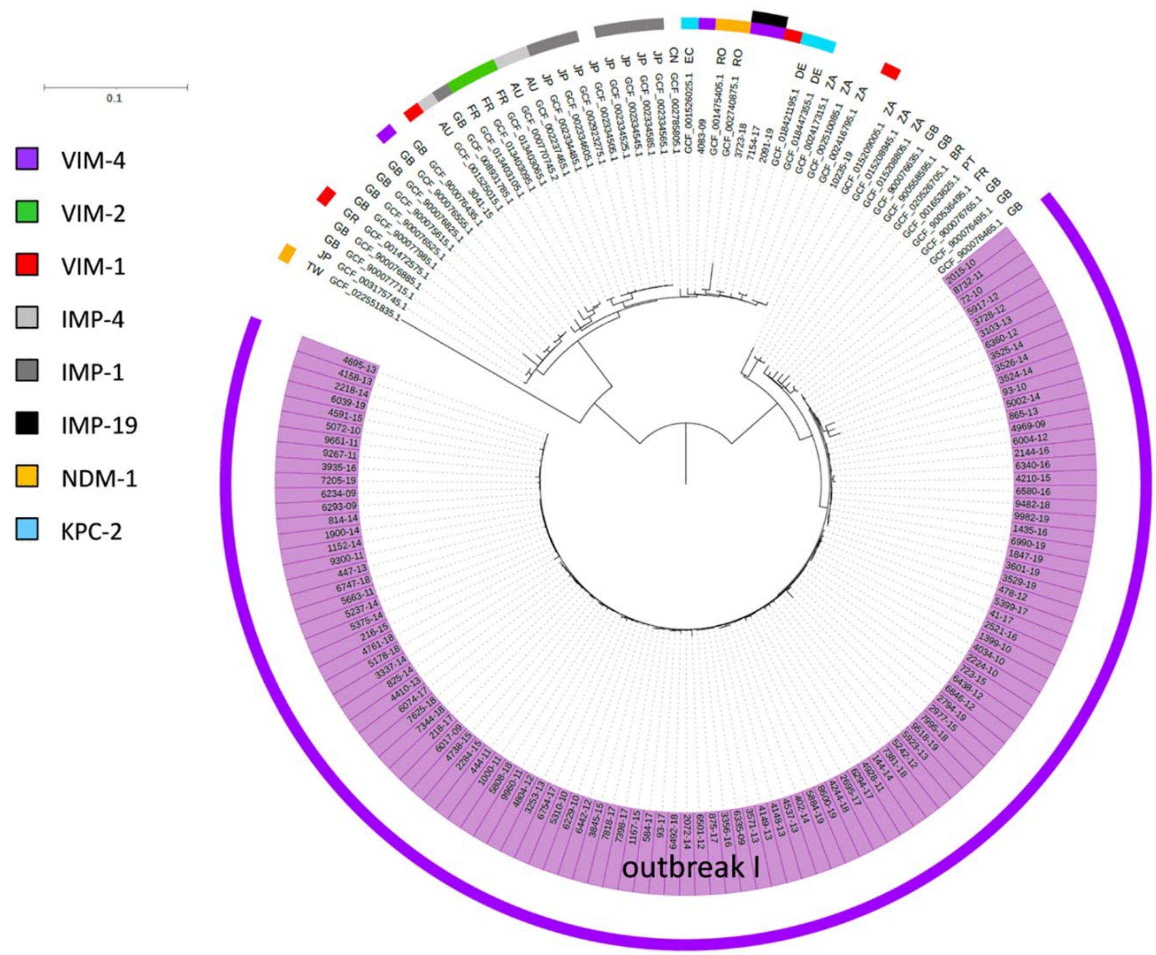

Appendix Figure 6. SNP-based phylogenetic tree of all study Polish *E. hormaechei* ST90 isolates compared with the international ST90 genomes available in GenBank. Numbers in the inner circle correspond to original numbers of the study isolates or GenBank assembly numbers. The presence of carbapenemases is indicated in the outer circles using corresponding colors. The country of origin other than Poland is presented with country codes: AU, Australia; CN, China; DE, Germany; EC, Ecuador; FR, France; GB, Great Britain; JP, Japan; PT, Portugal; RO, Romania; TW, Taiwan; ZA, South Africa. The tree was constructed using Parsnp and visualized with iTOL.

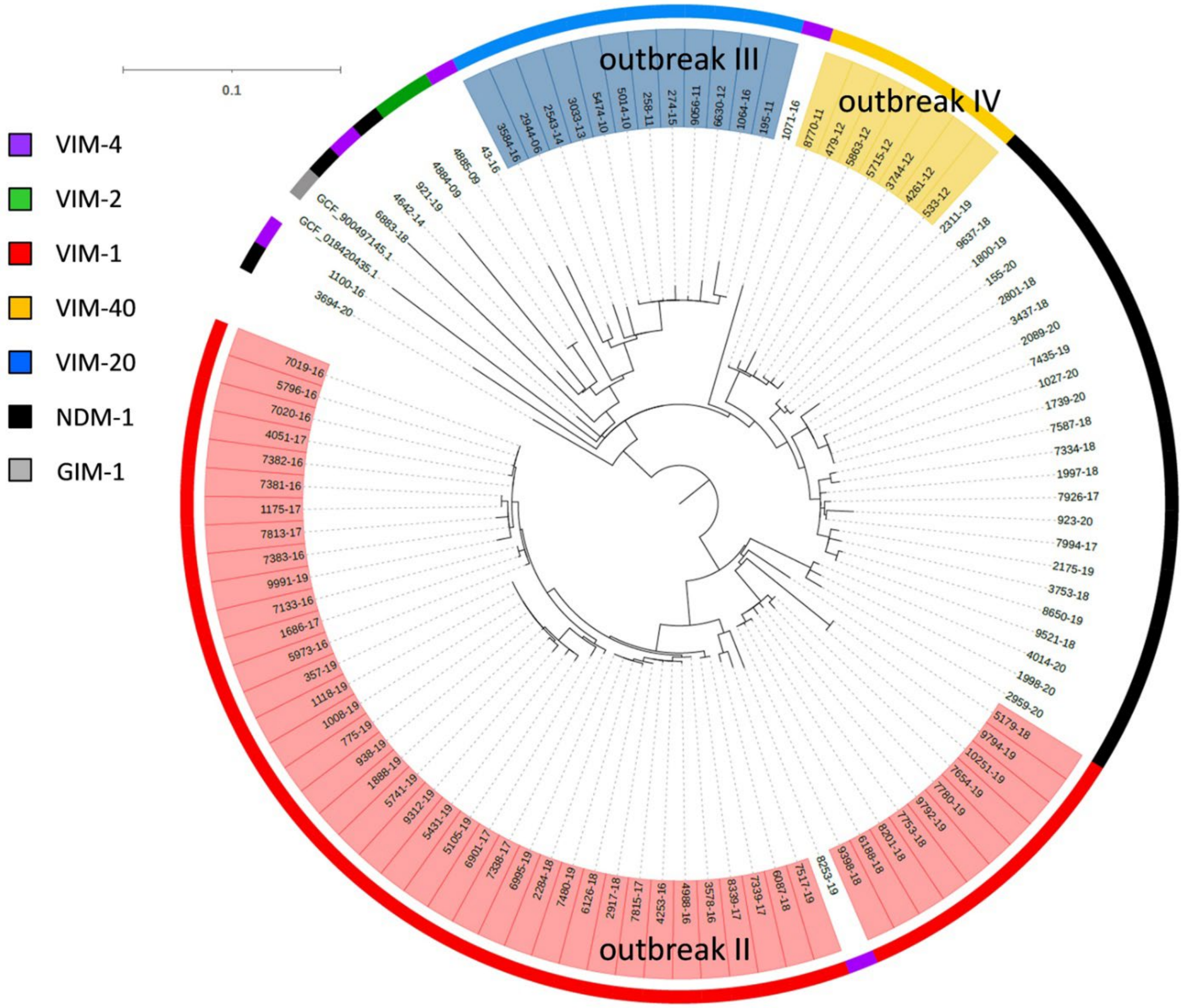

Appendix Figure 7. SNP-based phylogenetic tree of all study Polish *E. hormaechei* ST89 isolates compared with the international ST89 genomes available in RefSeq. Numbers in the inner circle correspond to original numbers of the study isolates or RefSeq assembly numbers. The presence of carbapenemases is indicated in the middle circle using corresponding colors. The region of Poland where isolates were recorded is presented in outer circle. DE, Germany. The tree was constructed using Parsnp and visualized with iTOL.

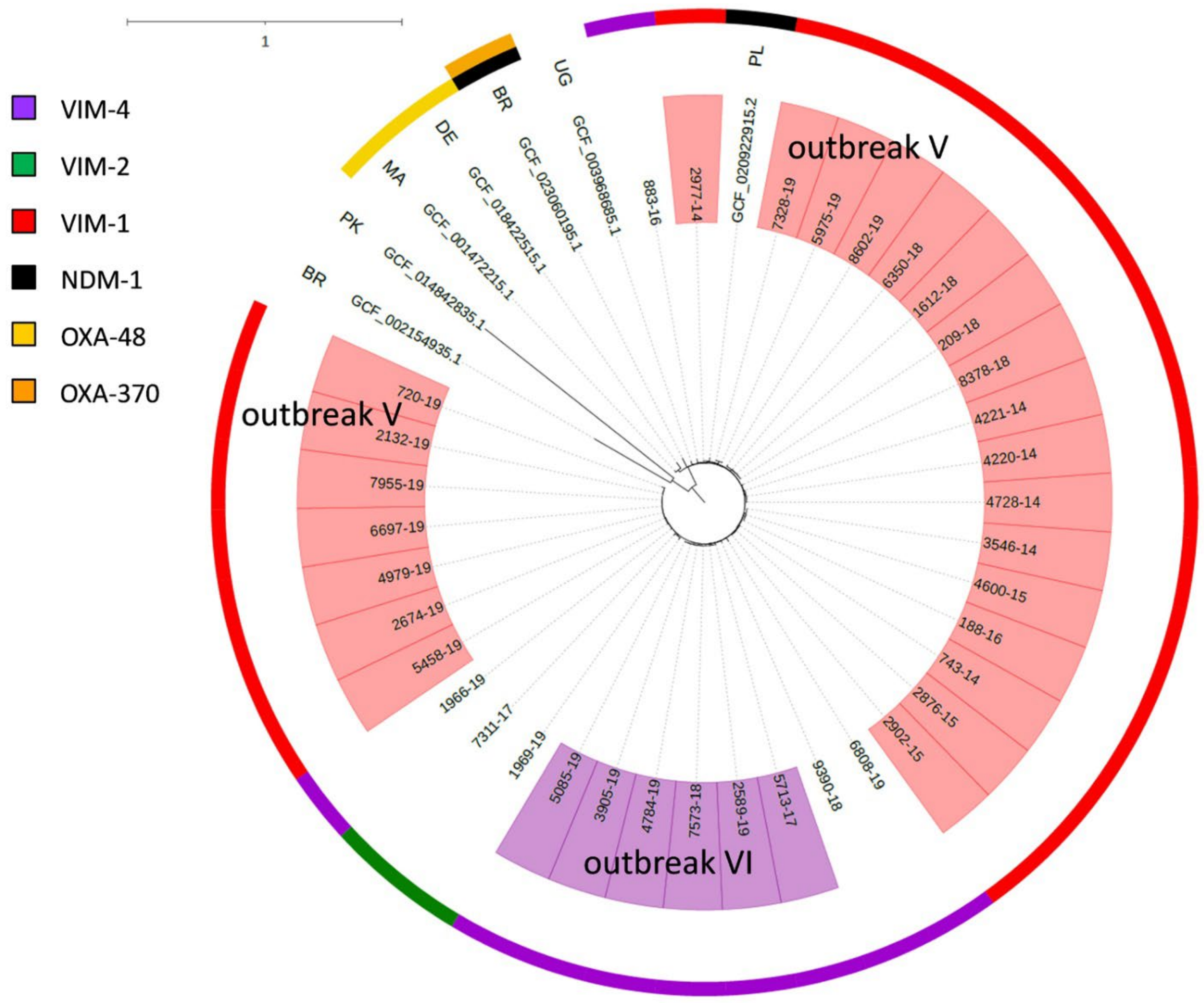

Appendix Figure 8. SNP-based phylogenetic tree of all study Polish *E. hormaechei* ST121 isolates compared with the international ST121 genomes available in RefSeq. Numbers in the inner circle correspond to original numbers of the study isolates or RefSeq assembly numbers. The presence of carbapenemases is indicated in the outer circles using corresponding colors. The country of origin other than studied isolates is presented with country codes: BR, Brazil; DE, Germany; MA, Morocco; PK, Pakistan; PL, Poland; UG, Uganda. The tree was constructed using Parsnp and visualized with iTOL.

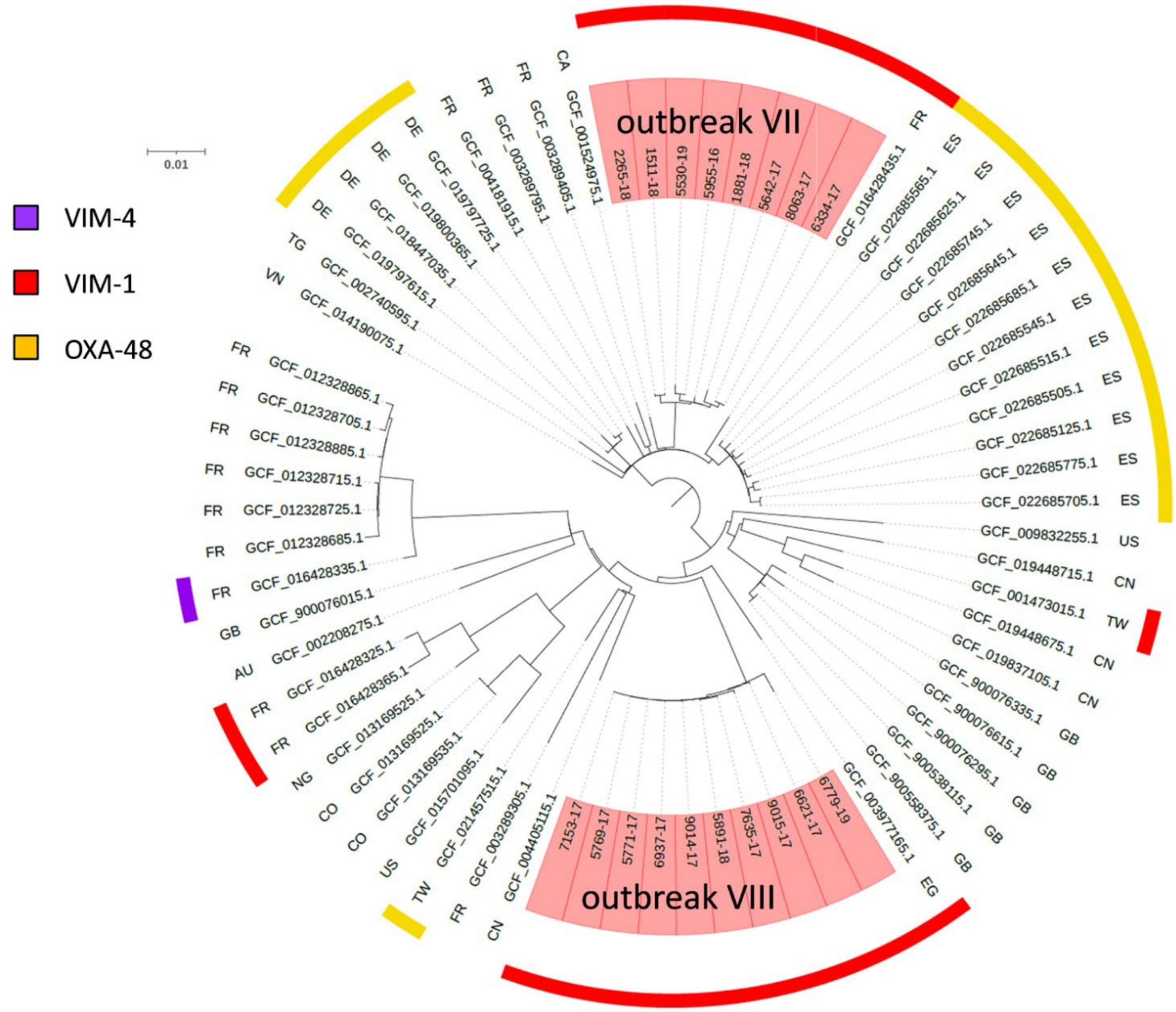

Appendix Figure 9. SNP-based phylogenetic tree of all study Polish *E. hormaechei* ST66 isolates compared with the international ST66 genomes available in RefSeq. Numbers in the inner circle correspond to original numbers of the study isolates or RefSeq assembly numbers. The presence of carbapenemases is indicated in the outer circles using corresponding colors. The country of origin other than Poland is presented with country codes: AU, Australia; CA, Canada; CN, China; CO, Colombia; DE, Germany; EG, Egypt; ES, Spain; FR, France; GB, Great Britain; NG, Nigeria; TW, Taiwan; TG, Togo; US, United States; VN, Vietnam. The tree was constructed using Parsnp and visualized with iTOL.

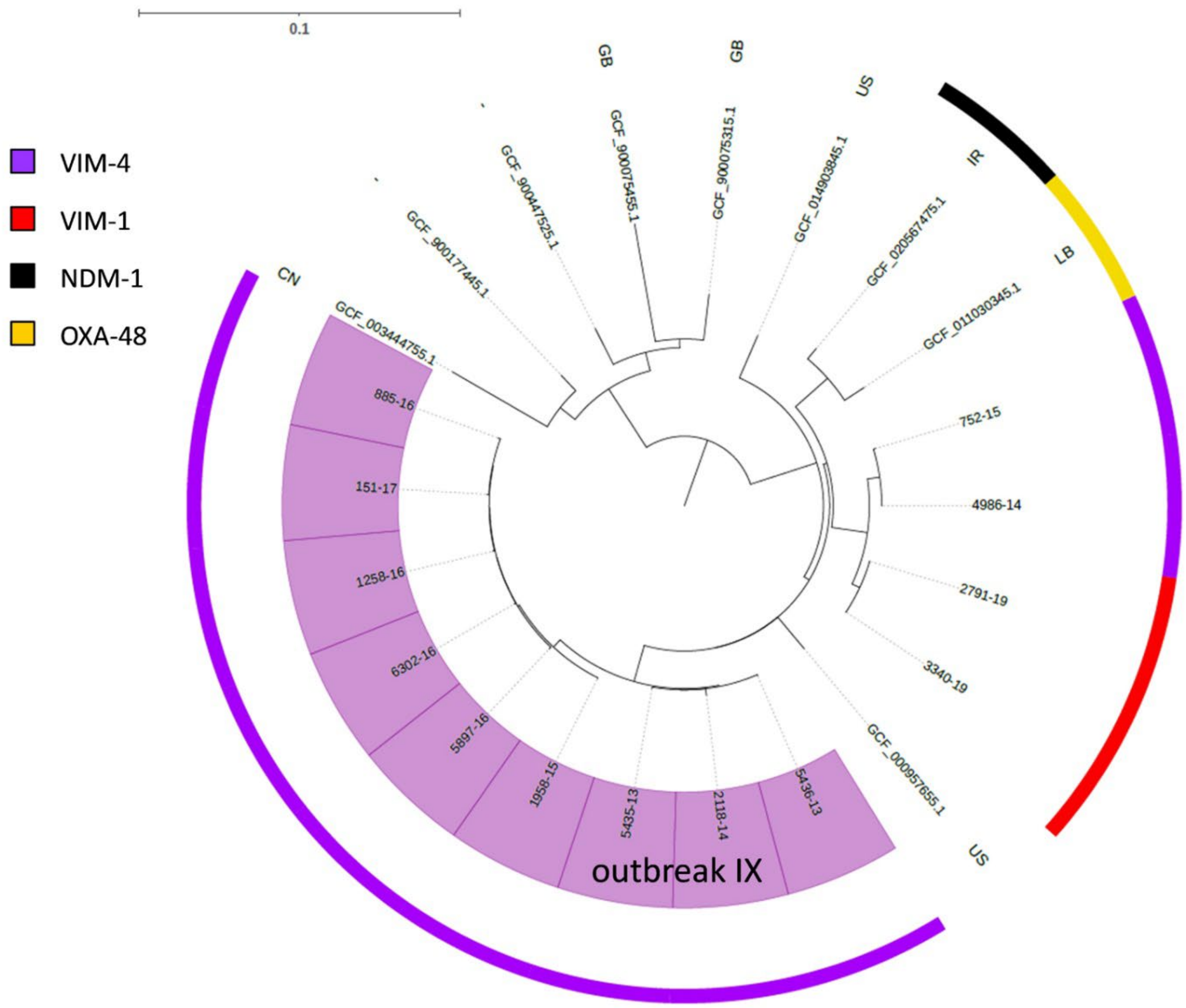

Appendix Figure 10. SNP-based phylogenetic tree of all study Polish *E. hormaechei* ST134 isolates compared with the international ST134 genomes available in RefSeq. Numbers in the inner circle correspond to original numbers of the study isolates or RefSeq assembly numbers. The presence of carbapenemases is indicated in the outer circles using corresponding colors. The country of origin other than Poland is presented with country codes: CN, China; GB, Great Britain; IR, Iran; LB, Lebanon; US, United States. The tree was constructed using Parsnp and visualized with iTOL.

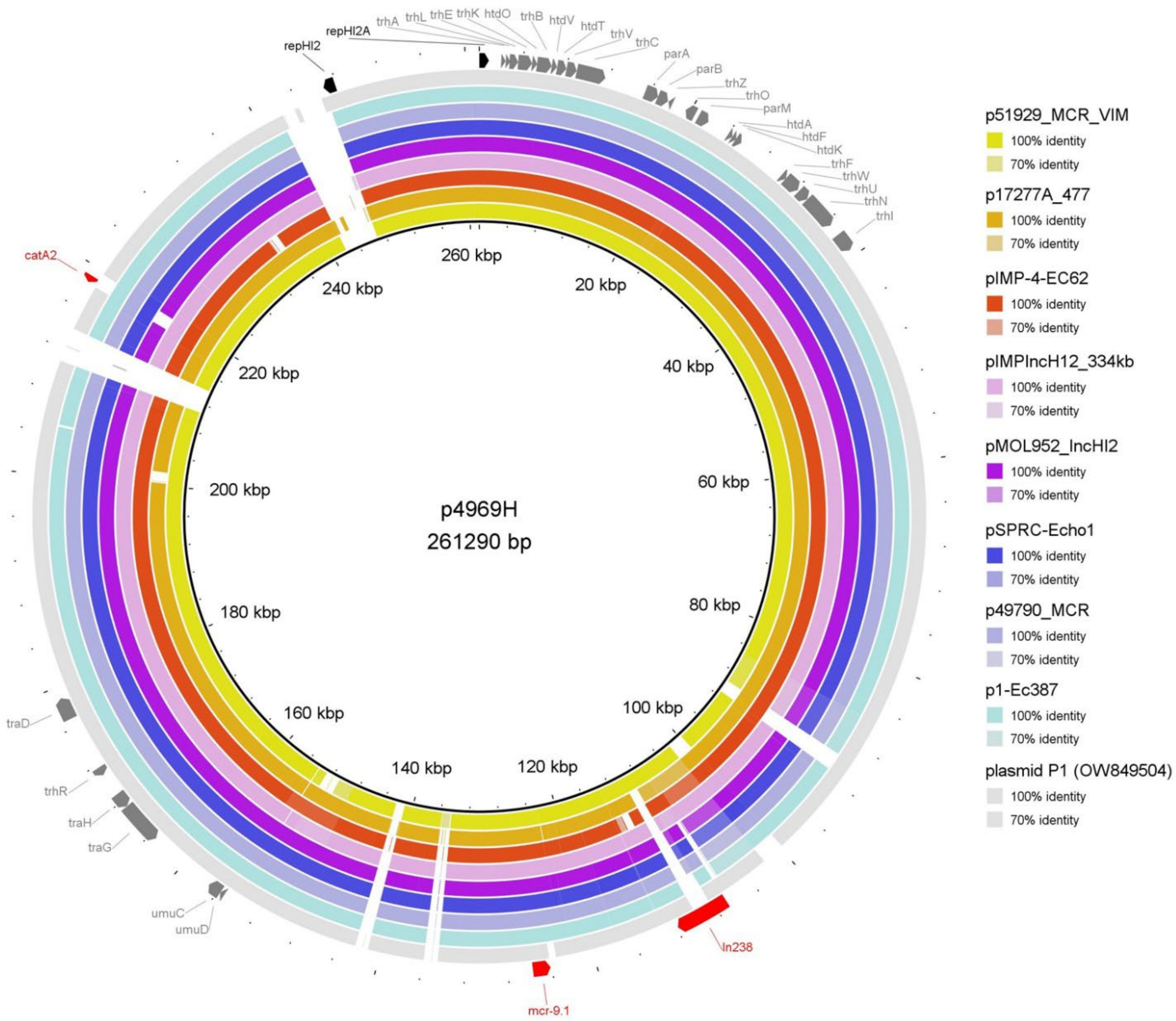

Appendix Figure 11. Comparison of the VIM-4-encoding (integron In238) IncHI2+HI2A p4969H plasmid (inner, thin black circle) to previously reported plasmids of the highest homology: p17277A_477 (Argentina; CP043927), pIMP-4-EC62 (China; MH829594), pIMPIncHI2_334kb (United Kingdom; CP044215), pMOL952_IncHI2 (Italy; OU015717), pSPRC-Echo1 (Australia; CP032842), p49790_MCR (Czechia; CP059425), p1-Ec387 (Japan; AP024583) and P1 (Spain; OW849504). The outer thick black ring refers to the annotation of p4969H, with the selected genes indicated. The percentage of sequence identity is reflected by color intensity. The picture was created using BRIG software (http://brig.sourceforge.net/).

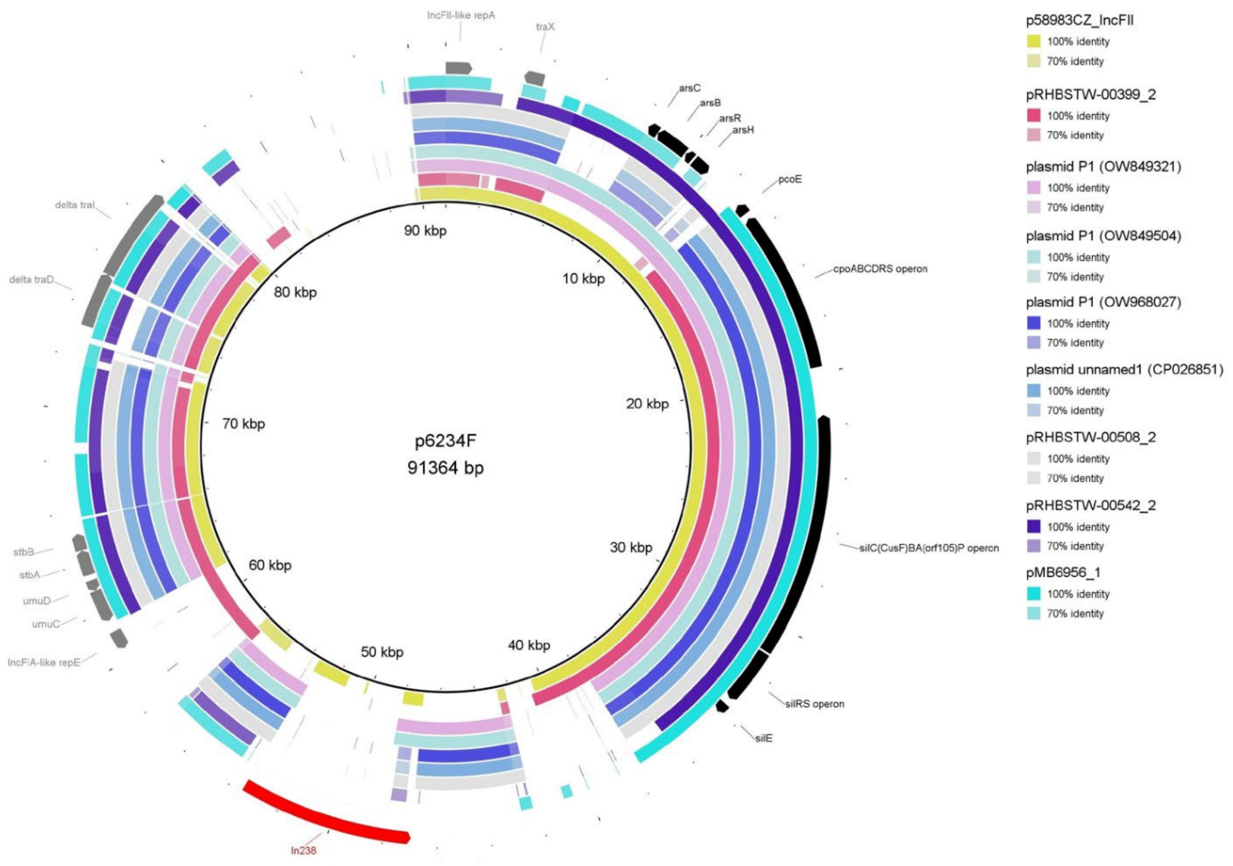

Appendix Figure 12. Comparison of the VIM-4-encoding (integron In238) IncFII+FIA p6234F plasmid (inner, thin black circle) to previously reported plasmids of the highest homology: p58983CZ_IncFII (Czechia; CP085735), pRHBSTW-00399_2 (United Kingdom; CP056561), plasmid P1 (Spain; OW849321), plasmid P1 (Spain; OW849504), plasmid P1 (Spain; OW968027), plasmid unnamed1 (United States; CP026851), pRHBSTW-00508_2 (United Kingdom; CP056442), pRHBSTW-00542_2 (United Kingdom; CP056712), pMB6956_1 (United States; CP103612)**.** The outer thick black ring refers to the annotation of p6234F, with the selected genes indicated. The percentage of sequence identity is reflected by color intensity. The picture was created using BRIG software (http://brig.sourceforge.net/).

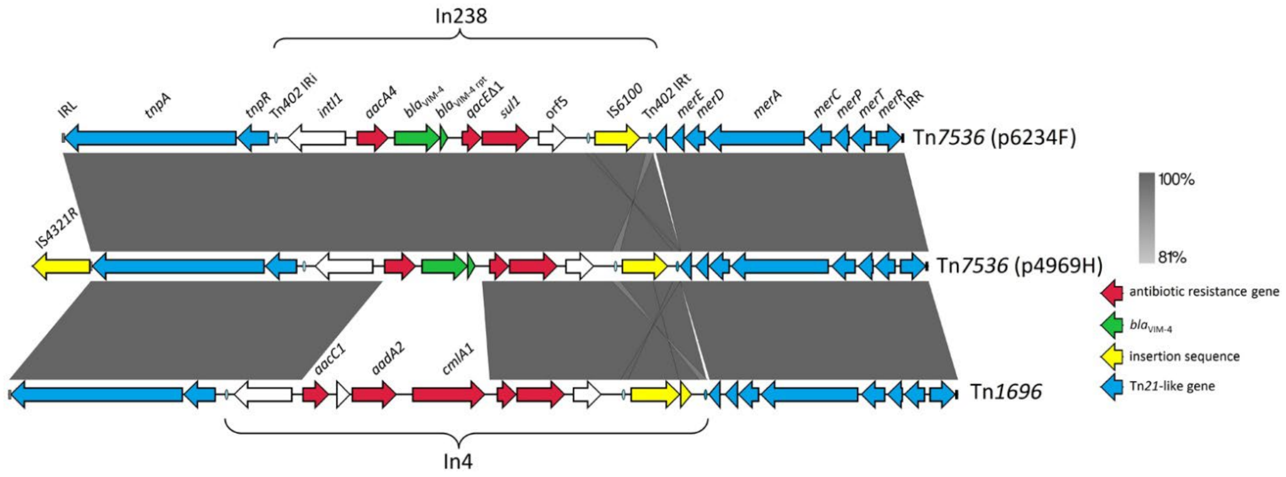

Appendix Figure 13. Comparison of sequences of the In238-containing Tn*21*-like elements, Tn*7536*, from p6234F and p4969H, and the most similar transposon Tn*1696* with the In4 integron (U12338). The percentage of sequence identity is reflected by gray color intensity. Individual loci (antibiotic resistance, mobile genetic elements, heavy metal resistance genes) are marked by colored arrows or triangles as explained below. The picture was created using the Easyfig 2.2.5 software.

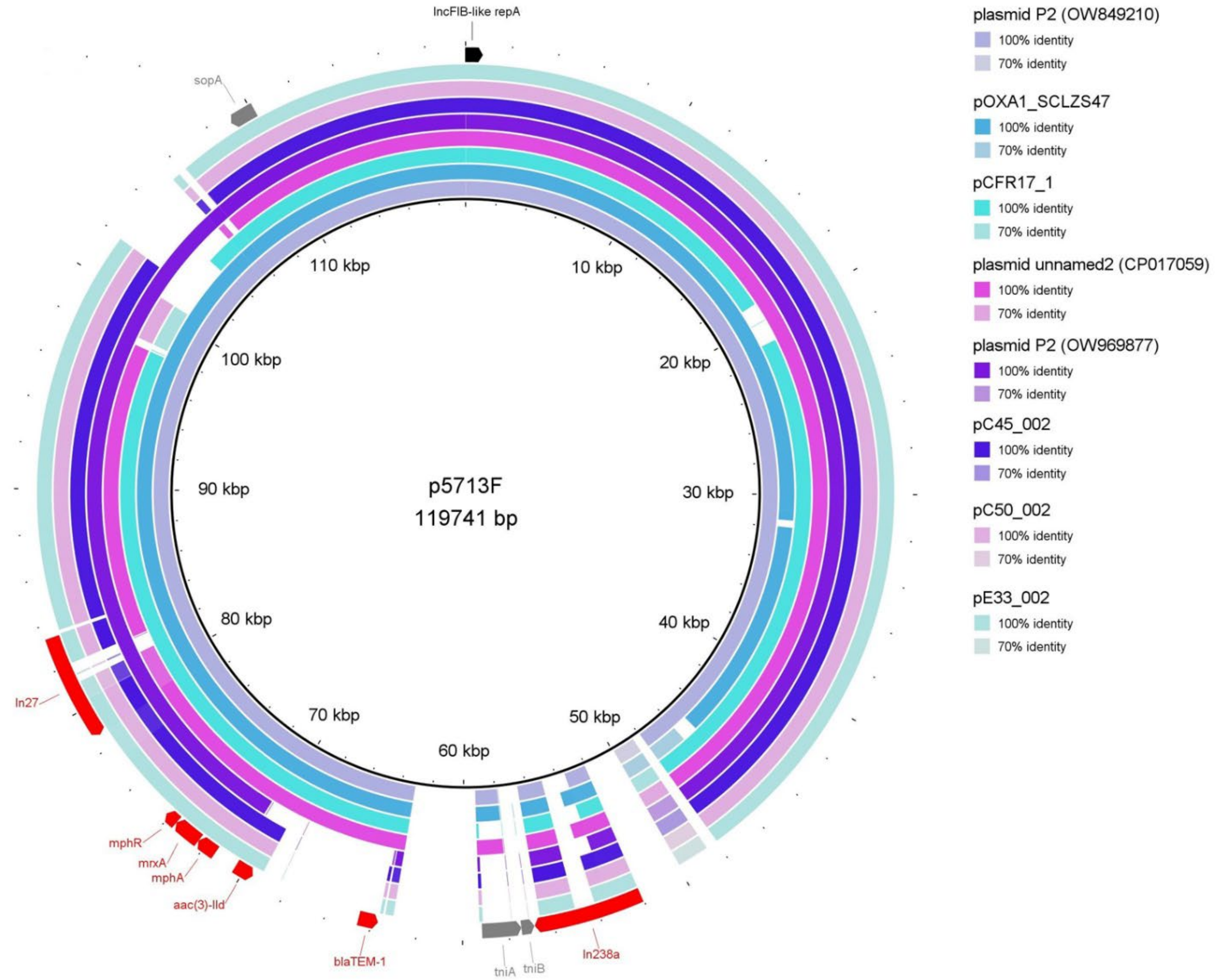

Appendix Figure 14. Comparison of the VIM-4-encoding (integron In238a) IncFIB p5713F plasmid (inner, thin black circle) to previously reported plasmids of the highest homology: P2 (Spain; OW849210), pOXA1_SCLZS47 (China; CP092496), pCFR17_1 (China; CP035277), plasmid unnamed2 (Sierra Leone; CP017059), plasmid P2 (Spain; OW969877), pC45_002 (Australia; CP042553), pC50_002 (Australia; CP042480) and pE33_002 (Australia; CP042519). The outer thick black ring refers to the annotation of p5713F, with the selected genes indicated. The percentage of sequence identity is reflected by color intensity. The picture was created using BRIG software (http://brig.sourceforge.net/).

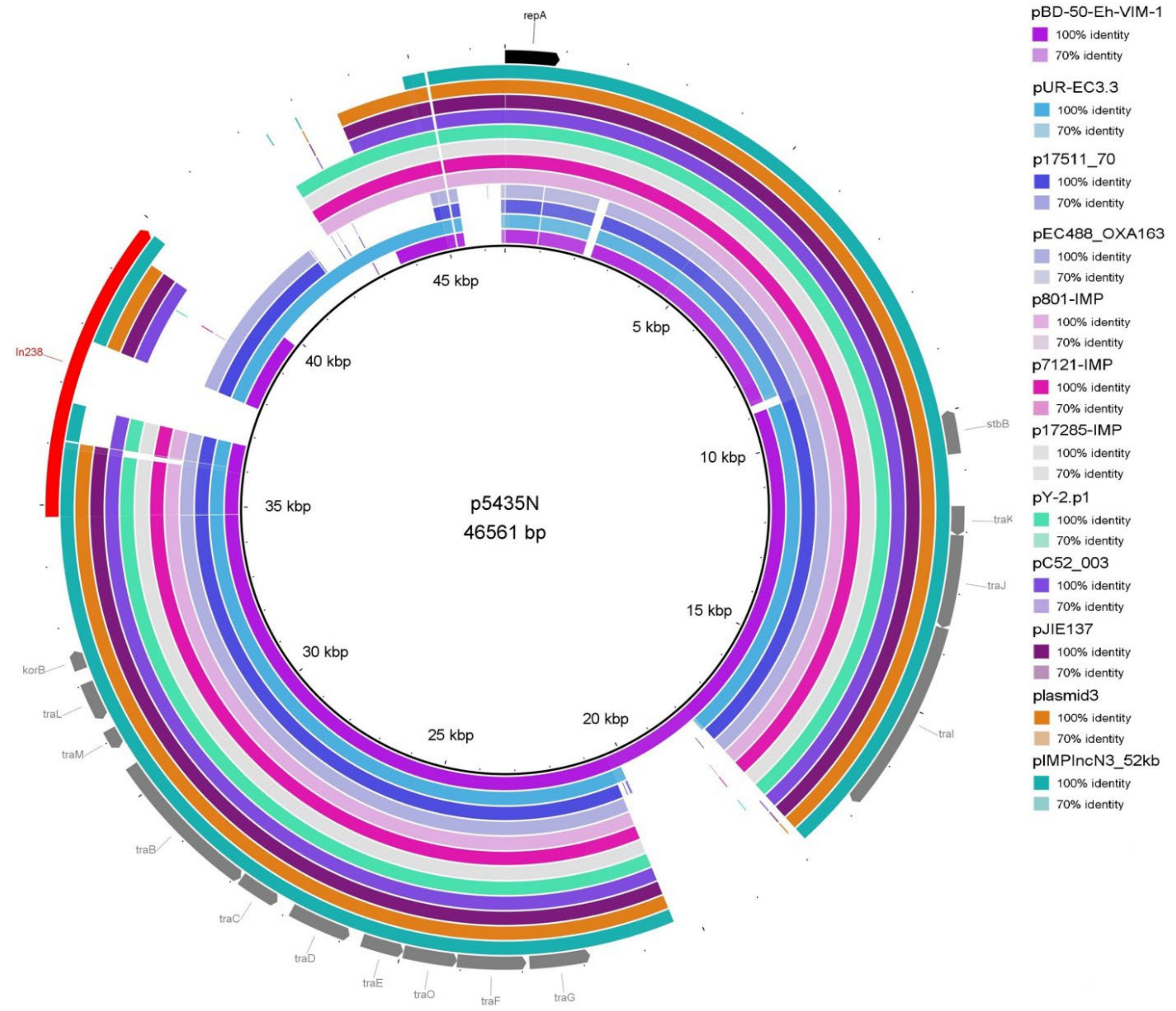

Appendix Figure 15. Comparison of the VIM-4-encoding (integron In238) IncN p5435N plasmid (inner, thin black circle) to previously reported plasmids of the highest homology: pBD-50-Eh-VIM-1 (Switzerland; CP063227), pUR-EC3.3 (Uruguay; MZ382872), p17511_70 (Argentina; MN583554), pEC448_OXA163 (Argentina; CP015078), p0801-IMP (China; KT345947), p7121-IMP (China; KX784502), p17285-IMP (China; KX784503), pY-2.p1 (China; CP090797), pC52_003 (Australia; CP042548), pJIE137 (Australia; EF219134), plasmid:3 (Australia; LR890351), pIMPIncN3_52kb (United Kingdom; CP043856). The outer thick black ring refers to the annotation of p5435N, with the selected genes indicated. The percentage of sequence identity is reflected by color intensity. The picture was created using BRIG software (http://brig.sourceforge.net/).

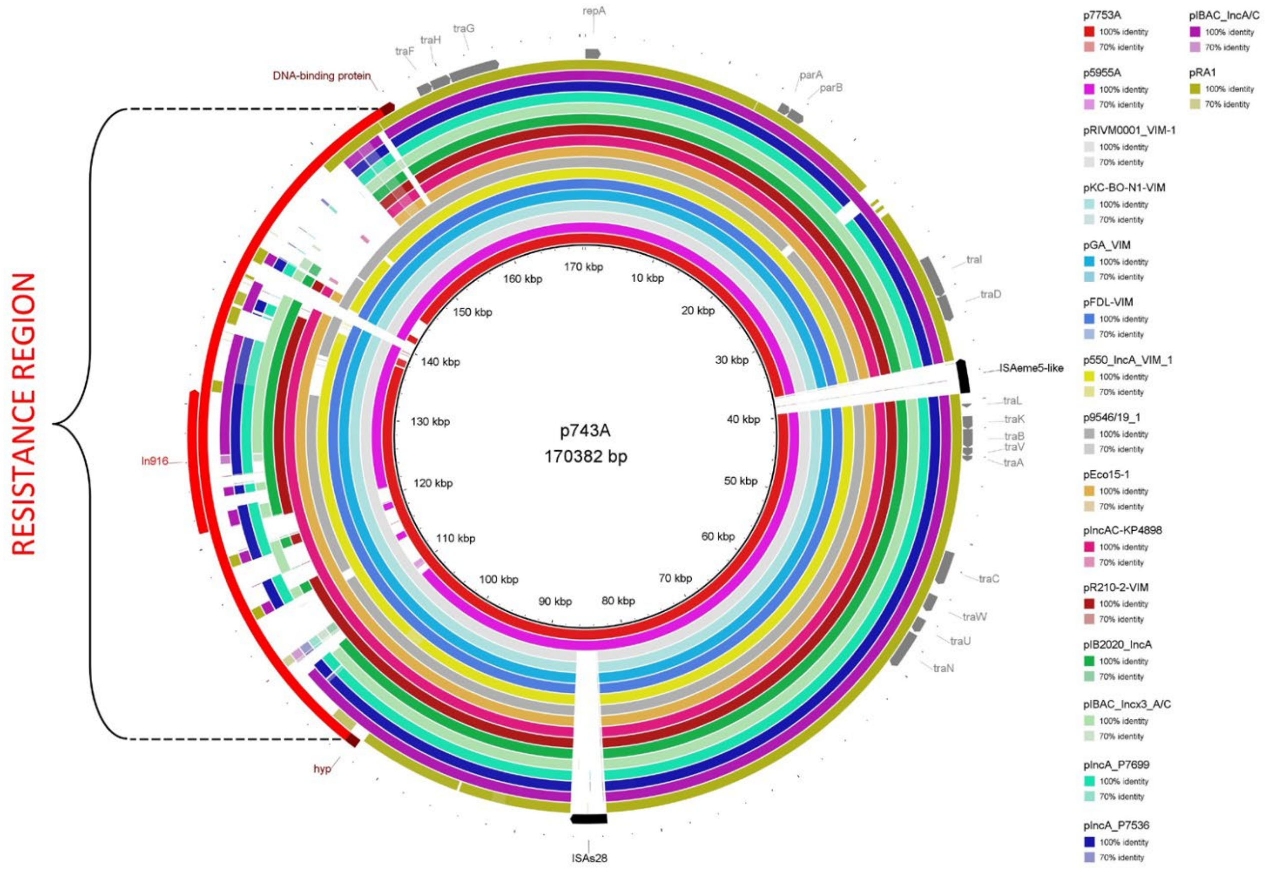

Appendix Figure 16. Comparison of the VIM-1-encoding (integron In916) IncA p743A plasmid (inner, thin black circle) to p7753A, p5955A, and previously reported plasmids of the highest homology: pRIVM0001_VIM-1 (The Netherlands; MH220284), pKC-BO-N1-VIM (Italy; MG228427), pGA_VIM (Italy; MN783743), pFDL-VIM (Italy; MN783744), p550_IncA_VIM_1 (Italy; CP058224), p9546/19_1 (Poland; ON081626), pEco15-1 (Canada; CP047711), pIncAC-KP4898 (Italy; KY882285), pR210-2-VIM (China; CP034084), pIB2020_IncA (Italy; CP059480), pIBAC_Incx3_A/C (Italy; MH594478), pIncA_P7699 (France; CP071911), pIncA_P7536 (France; CP071789), pIBAC_IncA/C (Italy; MH594477), pRA1 (United States; FJ705807). The outer rings refers to the annotation of p743A, with the selected genes indicated. The percentage of sequence identity is reflected by color intensity. The picture was created using BRIG software (http://brig.sourceforge.net/).

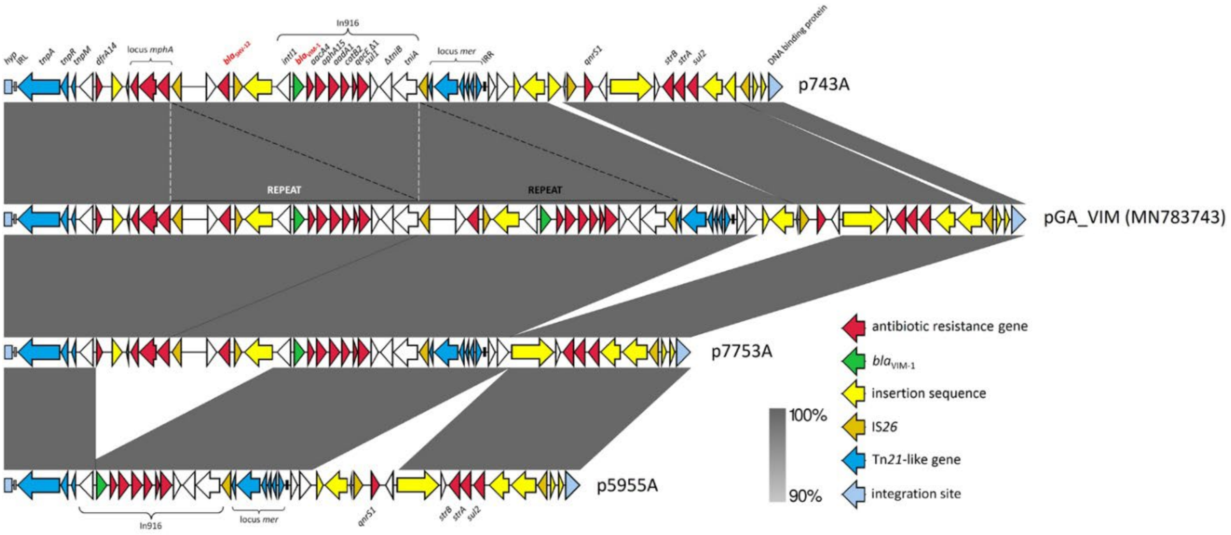

Appendix Figure 17. Comparison of the resistance regions of In916-carrying IncA-like plasmids characterized by MinION sequencing in the study (p743A, p7753A and p5955A) with the corresponding part of the pGA_VIM plasmid reported in Italy (MN783743). The percentage of sequence identity is reflected by gray color intensity. Individual loci (antibiotic resistance genes, mobile genetic elements, heavy metal resistance genes and integration sites) are marked by colored arrows or triangles as explained below. The picture was created using the Easyfig 2.2.5 software.

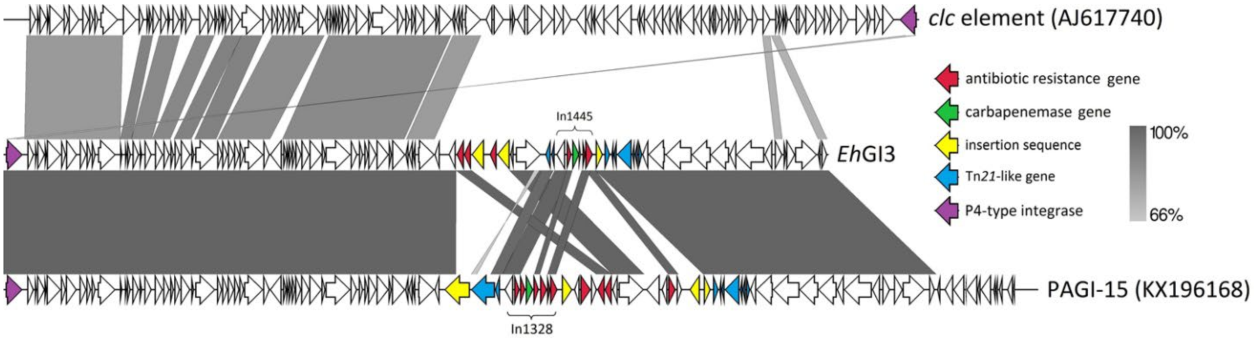

Appendix Figure 18. Comparison of the *Eh*GI3 sequence with the reference *clc*-type element (GenBank acc. No. AJ617740; Gaillard M, et al. J Bacteriol. 2006; 188:1999-2013) and the GI with the highest homology PAGI-15 (GenBank acc. No. KX196168; Hong JS, et al. Antimicrob Agents Chemother. 2016; 60:7216-23). The percentage of sequence identity is reflected by gray color intensity. Individual loci (antibiotic resistance, mobile genetic elements, heavy metal resistance genes, integrase genes) are marked by coloured arrows or triangles as explained below. The picture was created using the Easyfig 2.2.5 software.
